# Metabolomics Analysis and Biochemical Profiling of Arsenic-Induced Metabolic Impairment and Disease Susceptibility

**DOI:** 10.3390/biom13091424

**Published:** 2023-09-20

**Authors:** Syed Muhammad Shoaib, Samina Afzal, Ali Feezan, Muhammad Sajid Hamid Akash, Ahmed Nadeem, Tahir Maqbool Mir

**Affiliations:** 1 Department of Pharmaceutical Chemistry, Bahauddin Zakariya University, Multan 60800, Pakistan; 2 Department of Pharmaceutical Chemistry, Government College University, Faisalabad 38000, Pakistan; 3 Department of Pharmacology and Toxicology, College of Pharmacy, King Saud University, Riyadh 11451, Saudi Arabia; 4 National Center for Natural Products Research, School of Pharmacy, University of Mississippi, Oxford, MS 38677, USA

**Keywords:** ICP-MS, lipid metabolomes, amino acid metabolomes, arsenic metabolites, tandem mass spectrophotometer (MS/MS)

## Abstract

Our study aimed to conduct a comprehensive biochemical profiling and metabolomics analysis to investigate the effects of arsenic-induced metabolic disorders, with a specific focus on disruptions in lipid metabolism, amino acid metabolism, and carbohydrate metabolism. Additionally, we sought to assess the therapeutic potential of resveratrol (RSV) as a remedy for arsenic-induced diabetes, using metformin (MF) as a standard drug for comparison. We measured the total arsenic content in mouse serum by employing inductively coupled plasma mass spectrometry (ICP-MS) after administering a 50-ppm solution of sodium arsenate (50 mg/L) in purified water. Our findings revealed a substantial increase in total arsenic content in the exposed group, with a mean value of 166.80 ± 8.52 ppb (*p* < 0.05). Furthermore, we investigated the impact of arsenic exposure on various biomarkers using enzyme-linked immunosorbent assay (ELISA) methods. Arsenic exposed mice exhibited significant hyperglycemia (*p* < 0.001) and elevated levels of homeostatic model assessment of insulin resistance (HOMA-IR), hemoglobin A1c (Hb1Ac), Inflammatory biomarkers as well as liver and kidney function biomarkers (*p* < 0.05). Additionally, the levels of crucial enzymes linked to carbohydrate metabolism, including α-glucosidase, hexokinase, and glucose-6-phosphatase (G6PS), and oxidative stress biomarkers, such as levels of glutathione (GSH), glutathione reductase (GR), glutathione peroxidase (GPx), catalase, and superoxide dismutase (SOD), were significantly reduced in the arsenic-exposed group compared to the control group (*p* < 0.05). However, the level of MDA was significantly increased. Molecular analysis of gene expression indicated significant upregulation of key enzymes involved in lipid metabolism, such as carnitine palmitoyl-transferase-I (CPT-I), carnitine palmitoyl-transferase-II (CPT-II), lecithin–cholesterol acyltransferase (LCAT), and others. Additionally, alterations in gene expression related to glucose transporter-2 (GLUT-2), glucose-6-phosphatase (G6PC), and glucokinase (GK), associated with carbohydrate metabolism, were observed. Amino acid analysis revealed significant decreases in nine amino acids in arsenic-exposed mice. Metabolomics analysis identified disruptions in lipid metabolomes, amino acids, and arsenic metabolites, highlighting their involvement in essential metabolic pathways. Histopathological observations revealed significant changes in liver architecture, hepatocyte degeneration, and increased Kupffer cells in the livers of arsenic-exposed mice. In conclusion, these findings enhance our comprehension of the impact of environmental toxins on metabolic health and offer potential avenues for remedies against such disruptions.

## 1. Introduction

Arsenic, a toxic metalloid widely distributed throughout the Earth’s crust, comprises various allotropic forms and constitutes a major component of over 200 mineral species [1]. Geologists have identified arsenic as the 20th most abundant element in the Earth’s crust, with an average concentration of 2 mg/kg [2,3]. Arsenic exists in both inorganic forms (such as arsenic trioxide, sodium arsenite, sodium arsenate, and arsenic trichloride) and organic forms (including arsanilic acid, methyl-arsonic acid, dimethyl-arsinic acid, and arseno-betaine), present in the environment. Generally, organic forms are less toxic than their inorganic counterparts [4]. In its natural state, arsenic exists in four oxidation states: As(V), As(III), As(0), and As(III) [5]. Arsenopyrite, a mineral complex containing arsenic, iron, and sulfur, is the most frequently occurring form of arsenic in the environment [6]. Various sources contribute to arsenic exposure, including groundwater with solubilized arsenic. Natural and anthropogenic activities are considered the primary contributors to the increased contamination of drinking water with arsenic. Activities such as mineral extraction, the utilization of pesticides and fertilizers, the incorporation of additives in poultry feed, and waste processing have been identified as sources of drinking water contamination [7,8,9].

In groundwater, arsenic exists in both trivalent and pentavalent forms [9]. The toxicity of trivalent arsenicals is more pronounced than that of pentavalent arsenicals, leading to chronic arsenicosis in developing countries, including China, Pakistan, Bangladesh, Nepal, Iran, and India, as well as Chile and Taiwan [10]. The maximum allowable limit of arsenic in drinking water, established by the WHO, US-EPA, and EU, is 10 ppb [11,12,13]. Both acute and chronic exposure to arsenic through drinking water can be considered risk factors for various human diseases, including skin cancer, skin pigmentation changes, hyperkeratosis, heart disease, hypertension, lung cancer, pulmonary disease, diabetes mellitus, liver cancer, cervical cancer, prostate cancer, bladder cancer, and upper urinary tract carcinoma [14,15]. Trivalent arsenicals are harmful because they bind to the thiol groups of many biologically active proteins, inhibiting numerous enzymes involved in carbohydrate metabolism and other metabolic processes [16,17]. This interference affects pathways such as glucose metabolism, protein metabolism, choline metabolism, and membrane phospholipid breakdown [18,19], ultimately impacting apoptosis and disrupting endocrine signaling, leading to metabolic disorders. Arsenic, along with other environmental toxins, contributes to oxidative stress in cells. An important indicator of oxidative damage, membrane phospholipid breakdown, and cellular injury is the measurement of lipid oxidation. Malondialdehyde (MDA) concentrations are frequently used as a biochemical biomarker of oxidative stress, reflecting the extent of lipid oxidation [17]. Chronic arsenic exposure has been associated with proteome and metabolome changes in both humans and animal models. While the precise mechanisms underlying these changes are not fully understood, they may be connected to disruptions of specific biological pathways through epigenetic modifications. Metabolomics studies are now widely utilized to explore the altered pathways contributing to the development of various diseases, particularly in chronic diseases and metabolic syndromes characterized by inflammation. In this context, research into lipid metabolism is crucial [20,21,22]. Adiponectin, an adipocyte-derived hormone, plays a vital role in improving insulin sensitivity and regulating lipid metabolism independently of insulin.

Diabetes Mellitus (DM) occurs due to inadequate utilization of insulin or insufficient insulin production caused by pancreatic injury or disease, resulting in reduced insulin availability [23]. An autoimmune reaction against the proteins produced by β-islet cells of the pancreas is a major cause of type 1 diabetes mellitus (T1DM), while type 2 diabetes mellitus (T2DM) may arise from insulin resistance, impaired insulin secretion, and lifestyle habits [24]. Reportedly, arsenic-contaminated water leads to injury and apoptosis of the beta-cells of the pancreas, disrupting insulin production and function, ultimately resulting in insulin-dependent diabetes mellitus. Additionally, arsenic influences the expression of genes associated with insulin resistance, causing cells to deviate from their normal differentiation pathway and shift towards the proliferation pathway, which affects the sensitivity of peripheral tissues to insulin [25].

Resveratrol (3,5,4′-trihydroxy-trans-stilbene), a non-flavonoid polyphenolic molecule consisting of two phenol rings connected by an ethylene bridge, is synthesized in many plants as a response to injury. It was initially isolated in 1940 from white hellebore (Veratrum grandiflorum O. Loes) roots and subsequently in 1963 from Polygonum cuspidatum roots. Resveratrol is utilized as an anti-inflammatory, antioxidant, and anti-platelet agent in traditional Chinese and Japanese Medicine. An analogue of resveratrol with methoxylation (Pterostilbene), extracted from Pterocarpus santalinus (red sandalwood), is used in traditional medicine to treat diabetes. Pterostilbene activates AMPK, contributing to the maintenance of low blood sugar levels [26].

The primary objective of this study was to comprehensively investigate the intricate biochemical and metabolomic alterations induced by arsenic exposure and their subsequent impact on various metabolic pathways, specifically focusing on lipid, carbohydrate, and amino acid metabolism. The study aimed to uncover the underlying mechanisms by which arsenic disrupts these critical metabolic processes, contributing to the development of metabolic disorders, particularly diabetes mellitus. Additionally, the research sought to evaluate the potential therapeutic efficacy of resveratrol in alleviating the adverse effects of arsenic intoxication on metabolic health. By conducting a detailed analysis of biochemical biomarkers, gene expression profiles, and metabolomics data, the study aimed to provide a comprehensive understanding of the molecular intricacies associated with arsenic-induced metabolic perturbations and their potential amelioration through resveratrol intervention. Ultimately, the study aimed to contribute valuable insights into the complex interplay between environmental toxins, metabolic disruptions, and potential remedies, with implications for enhancing our knowledge of metabolic disorders and advancing therapeutic strategies for mitigation of their impact.

## 2. Materials and Methods

### 2.1. Preparation of Arsenic Solution

The 50-ppm sodium arsenate solution was prepared by dissolving 5 mg of sodium arsenate (Sigma-Aldrich, Saint Louis, MO, USA) in 100 mL of purified water. A fresh solution was prepared every three days for the duration of the study.

### 2.2. Preparation of Resveratrol Solution

Resveratrol (Anhui Min-metals Development Imp. & Exp. Co., Ltd. Hefei, China) is available in a coarse powdered form with low solubility in water. As a result, it was dissolved in peanut oil and administered according to the body weight of the experimental animal.

### 2.3. Preparation of Metformin Solution

A Metformin solution was created by dissolving a 250 mg tablet in 1 L of purified water, and the concentration of metformin was subsequently adjusted based on the body weight of the experimental animal before administration.

### 2.4. Study Design—Animal Grouping, Dosing Schedule and Routine Monitoring

This study was conducted on 48 healthy male mice (Mus musculus strain Swiss Albino Inbred species) over a span of 28 days. The mice were acquired from the animal house of the Faculty of Pharmacy at Government College University Faisalabad, Pakistan, after receiving approval from the Ethical Committee of the same institution (GCUF). The mice were then randomly allocated into four groups (*n* = 12 each): the control group, the arsenic-exposed group, the arsenic-exposed group with standard treatment, and the arsenic-exposed group with resveratrol treatment. The experiment was conducted under controlled laboratory conditions, maintaining a temperature of 25 °C ± 5 °C and a relative humidity of 50% ± 15%. Prior to the commencement of the experiment, the mice were weighed and found to weigh between 25 g and 35 g. Throughout the study, the mice were provided with standard mice chow and water as needed on a daily basis.

The groups were defined as follows:

1. Group-1 (CONT): Control group, given purified water.
2. Group-2 (Na_3_AsO_4_): Arsenic-exposed group, administered a 50-ppm solution of sodium arsenate (50 mg/L) in purified water.
3. Group-3 (MFT + Na_3_AsO_4_): Arsenic-exposed group treated with Metformin (250 mg/kg body weight/day).
4. Group-4 (RSV + Na_3_AsO_4_): Arsenic-exposed group treated with resveratrol in peanut oil at a dosage of 8 mg/kg body weight/day.

Throughout the study, the body weight and serum glucose levels of all mice were measured on the 1st, 7th, 14th, 21st, and 28th days.

### 2.5. Biochemical Analysis

At the conclusion of the dosing period, which spanned 28 days, the mice were sacrificed. Blood samples were collected from six mice within each group for the assessment of various biomarkers. These included markers related to glycemic control (serum glucose and insulin levels, HbA1c level, and insulin resistance HOMA-IR), carbohydrate metabolism (α-Amylase, hexokinase, α-glucosidase, and G6PC), liver function (ALT and AST), and kidney function (blood urea nitrogen (BUN) and creatinine). The blood samples from these mice were additionally subjected to ICP-MS analysis to determine the concentration of total arsenic.

For the remaining six mice within each group, blood samples were collected to analyze oxidative stress markers (glutathione (GSH), glutathione reductase (GR), glutathione peroxidase (GPx), catalase, superoxide dismutase (SOD), and malondialdehyde (MDA), as well as inflammatory biomarkers (C-reactive protein (CRP) and IL-6). The blood from these mice underwent amino acid analysis using an amino acid analyzer. Furthermore, the liver from these mice was obtained for gene expression analysis through qRT-PCR and histopathological examination, aiming to explore the impact of arsenic intoxication. The qRT-PCR analysis focused on genes involved in lipid metabolism (LCAT, CROT, CPT-1, CPT-II, CACT), amino acid metabolism (MRT), and carbohydrate metabolism (GAPDH, GK, GLUT-2, and G-6-PASE). Subsequently, the blood samples exclusively from mice exposed to arsenic and mice exposed to arsenic while being treated with resveratrol underwent metabolomics analysis using ESI+-MS/MS.

### 2.6. Biomarker Analysis for Estimation of Metabolic Impairment

Estimation of weight: The weight of each mouse was measured on the 1st day, 7th day, 14th day, 21st day, and 28th day using an A & D weighing scale.

#### 2.6.1. Estimation of Glycemic Index Biomarkers

The blood glucose level was checked in all groups using an On-Call Plus glucometer at the start of the study and after the first week, second week, third week, and fourth week of exposure to sodium arsenate, aiming to establish a relationship with diabetes mellitus (DM). According to the manufacturer’s instructions, serum insulin levels were determined using a mouse insulin ELISA kit (Catalog Number: E-EL-M1382, Elabscience^®^). Homeostatic model assessment for insulin resistance (HOMA-IR) was calculated using the following formula based on the measured fasting insulin (μU) and glucose (mM) levels.

HOMA IR = Fasting insulin (μU) × Fasting glucose (mM)/22.5

The HbA1c ELISA kit (Catalog Number: MBS2024955) was employed to determine the HbA1c levels, following the instructions provided by the manufacturer.

#### 2.6.2. Estimation of Carbohydrate Metabolism Biomarkers

Enzymes that were anticipated to undergo changes due to arsenic exposure were assessed from serum samples using the ELISA method. The appropriate ELISA kits for mouse α-amylase (Catalog Number: MBS031961), hexokinase (Catalog Number: MBS260342), α-glucosidase (Catalog Number: MBS3805225), and G6PC (Catalog Number: MBS2515790) were utilized following the assay protocols provided by Mybiosource Inc. San Diego, CA, USA.

#### 2.6.3. Estimation of Oxidative Stress Biomarkers

The Elisa kit method was employed to determine the levels of antioxidant enzymes, including total glutathione (GSH), glutathione reductase (GR), glutathione peroxidase (GPx), catalase, and superoxide dismutase (SOD), in the control group, arsenic-exposed group, and the groups exposed to arsenic treated either with metformin or resveratrol. The serum level of malondialdehyde (MDA) was also quantified using a microplate ELISA reader (800TS-UV, BioTek Instruments, Winooski, VT, USA) at a wavelength of 532 nm, utilizing an MDA assay kit (Mybiosource, San Diego, CA, USA).

#### 2.6.4. Estimation of Liver Function Biomarkers

Serum concentrations of alanine transaminase (ALT) and aspartate transaminase (AST) were determined using the photometric method. The absorbance was measured at a wavelength of 340 nm using the Biolab-310 serum analyzer. The absorbance values were subsequently converted into their corresponding concentrations (U/L) by multiplying by the appropriate dilution factor.

#### 2.6.5. Estimation of Kidney Function Biomarker

In accordance with the guidelines outlined in the user handbook accompanying the creatinine assay kit, the serum levels of blood urea nitrogen (BUN) and creatinine were quantified utilizing the sandwich ELISA method.

#### 2.6.6. Estimation of Inflammatory Biomarkers

The levels of CRP and IL-6 in the serum were assessed using the sandwich ELISA method. Prior to use, all chemicals and reagents were allowed to equilibrate at 25 °C. The required reagents, standards, and samples were prepared, and the analyses were conducted following the standard operating procedures provided by the manufacturer (PARS Biochem, Nanjing, China).

### 2.7. Estimation of Arsenic by ICP-MS

**Figure 1:**
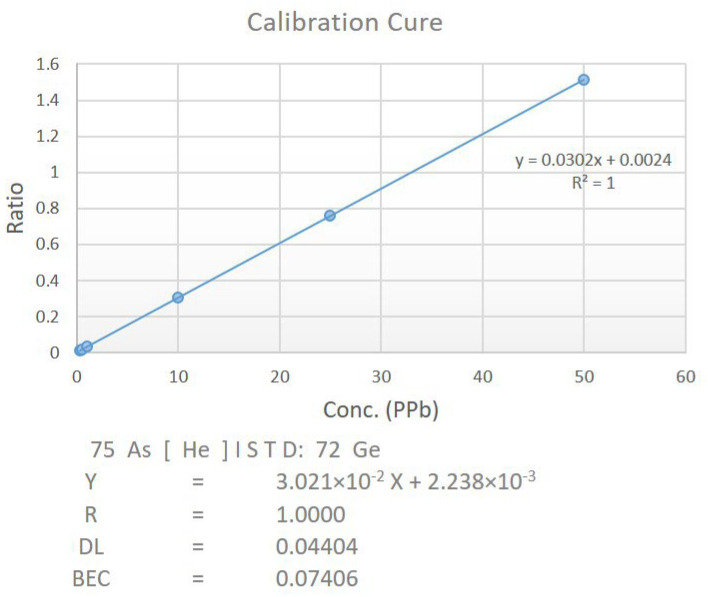
Calibration curve for ICP-MS. The x-axis represents the concentration of arsenic particles, while the y-axis depicts the ratio of the instrument’s measured arsenic particle count to its internal standard germanium particle count (CPS (75 As)/CPS (72 Ge)).

The treated blood samples were diluted 30 times using a gravimetric method with 2% nitric acid. Subsequently, these diluted samples were analyzed using an Agilent ICP-MS model number 7900. The analysis was conducted using the Mass Hunter 4.4 workstation software. The plasma torch was supplied with a flow of argon set between 1.2 to 1.7 L/min at 90 PSI. Additionally, a helium flow rate of 4.3 mL/min was directed to the collision cell. The sample uptake and stabilization parameters were set at 25 s and 20 s, respectively, for both calibration and sample analysis. Utilizing the atomizer, the samples and diluted standard solutions prepared with purified water were introduced into the plasma ion source of the ICP-MS. This ion source contains a high-energy argon plasma operating at a temperature of 6000–10,000 K. In this setup, liquid samples are transformed into aerosols, and charged ions are generated through the processes of evaporation, dissociation, and ionization. The ion acquisition system transports these charged ions into the mass spectrometer, where they are sorted and quantified based on their mass-to-charge (*m*/*z*) ratio within the mass analyzer. For calibration, a curve was generated as shown in Figure 1 using a series of arsenic standard solutions with concentrations of 0.300 ppb, 0.500 ppb, 1 ppb, 10 ppb, 15 ppb, 25 ppb, and 50 ppb.

### 2.8. Estimation of Gene Expression Metabolizing Enzymes

#### 2.8.1. RNA Isolation

For RNA isolation, approximately 100 mg of frozen liver was combined with 1 mL of TRIzol reagent (Biobasic BS410A-MA18DR0J) in an Eppendorf tube. The mixture was then centrifuged at 12,000× *g* for 10 min. The subsequent phase separation involved using chloroform and the supernatant from the first centrifugation. After vortexing the mixture for 15 s, it was incubated at 25–30 °C for 5–10 min. Following this incubation, the Eppendorf tubes were subjected to another centrifugation at 12,000× *g* for 15 min at 4 °C. This resulted in the contents of the tubes separating into two phases. The lower red phase consisted of the phenol-chloroform fraction, which separated from the upper aqueous phase containing RNA at the interface. The supernatant aqueous phase was carefully pipetted out without disturbing the interface, and it was transferred to respective pre-labeled tubes. The third step involved precipitating RNA using isopropyl alcohol. Subsequently, the RNA pellets were washed using a 75% ethanol solution and were then stored at −80 °C. For storage, 500 μL of RNA storing reagent was added to the samples.

#### 2.8.2. cDNA Synthesis

We utilized the Thermo Scientific RevertAid First Strand cDNA Synthesis Kit for conducting cDNA synthesis. Initially, we prepared the master mix for this process. The master mix, consisting of 20 µL, was composed of 5 µL of Template RNA (0.1 ng–5 µg), 1 µL of Oligo (dt18) primer, 6µL of Nuclease-free water, 4 µL of 5X reaction buffer, 1 µL of RiboLock RNase Inhibitor (20 U/µL), 2 µL of 10 mM dNTP Mix, and 1 µL of Revert Aid M-MuLV RT (200 U/µL). For each sample, 20 μL of the master mix was added to 5 µL of the previously extracted RNA sample. The mixture was gently mixed and then placed in a PCR machine for incubation at 42 °C for 60 min. Following the incubation, the reaction was terminated by heating at 70 °C for 5 min. This process yielded cDNA synthesis within an hour. The synthesized cDNA was stored at −20 °C for a short period, typically up to one week. For long-term storage, it was recommended to store the cDNA at −70 °C.

#### 2.8.3. qRT-PCR Procedure

Eurofins Genomics was engaged to design Custom DNA Oligos, which were supplied in an orange box. The dilution of the selected genes (as reported in Table 1) was carried out according to the manufacturer’s instructions using purified water, resulting in a concentration of 100 μM for each primer sequence (first dilution). The second dilution was created by taking 100 μL from the first dilution and further diluting it with 900 μL of purified water. Subsequently, a qRT-PCR plate was prepared by adding the second dilution of both the forward primer (1 μL) and reverse primer (1 μL), along with 5 μL of the cDNA sample and 5 μL of CYBR green into each well of the plate. Then, 2 μL of the previously prepared master mix was added to each well. The prepared plate underwent qRT-PCR using a standard protocol, involving thermal cycles starting with 95 °C for 10 min, followed by 40 cycles of denaturation at 95 °C for 15 s and annealing at 60 °C for 30 s. The resulting amplified PCR products were visualized using 2% agarose gel electrophoresis. The gene associated with lipid metabolism was β-actin, which served as an internal reference or housekeeping gene for normalizing the quantity of mRNA and complementary DNA. The GAPDH gene was used as a positive control for the genes (Glut-2, GK, and G6PH) involved in carbohydrate metabolism. To predict fold changes in mRNA expression for the selected genes in either an upregulation or downregulation form, the Livak method was employed.

### 2.9. Analysis of Amino Acid

Blood samples were collected in EDTA tubes during the time of mouse sacrifice. These samples were then subjected to centrifugation at 2000 rpm for 20 min at 4 °C, leading to the separation of plasma. The separated plasma was further centrifuged with a protein precipitating agent, typically 5% sulfosalicylic acid, at a 1:1 ratio. This was carried out at 10,000× *g* at 4 °C for 5 min, until a clear supernatant was achieved. On top of this clear supernatant, a lipid layer formed, which was meticulously removed. The supernatant was then dried using liquid nitrogen, preparing the sample for amino acid analysis. The operation of the amino acid analyzer was based on the principle of ion exchange chromatography with post-column derivatization using Ninhydrin. Calibration standards were prepared using amino acid standard solutions categorized as Acidics and Neutrals (A6407) and Basics (A1585), which were supplied by Sigma-Aldrich, Saint Louis, MO, USA. These standards were used to calibrate the amino acid analyzer.

### 2.10. Metabolomics Analysis

#### Qualitative Analysis of Metabolomes with MS/MS

Serum was obtained by centrifuging blood at 3500× *g* for 10 min at 4 °C. The obtained serum was stored at −80 °C in preparation for metabolomics analysis. For pretreatment, 200 μL of each serum sample was mixed with 600 μL of cold methanol by vigorous shaking. This mixture was stored for 10 min and subsequently centrifuged at 12,000× *g* for 10 min at 4 °C. The resulting supernatant from this centrifugation was filtered using a 0.22 μm PTFE filter. The filtered solution was then injected into an ion trap mass spectrometer for tandem mass spectroscopy, which was housed at the National Institute of Biotechnology and Genetic Engineering in Faisalabad, Pakistan. The instrument was operated with the following instrumental and operational parameters, as reported in Table 2. Data were acquired using both positive (ESI+) and negative (ESI−) electrospray ionization modes, with the capillary voltage set to 4.7 kV. The data acquisition spanned the *m*/*z* range from 50 to 2000 in MSE mode. The full *m*/*z* range spectrum would be divided into multiple segments with the scanning of each segment to produce multiple narrow-range spectra during the ms/ms data acquisition. In order to retrieve the peak pair information from these segmented spectra, each peak pair resulting from a differentially labelled metabolite in the analysis of the sample was processed independently. For fragmentation, several peaks were chosen and subjected to collision-induced dissociation (CID) with energy levels ranging from 20 to 30. This allowed for the identification of various metabolomes, including lipid metabolomes, amino acids, and arsenic metabolites.

### 2.11. Histopathological Assessment

Liver tissues were subsequently ready for histopathological evaluation. The tissues were processed using a standard tissue processor, undergoing dehydration through a sequence of alcohol concentrations: 70%, 95%, and finally absolute alcohol. Tissue clearing was achieved using a solution consisting of 50% xylene and 50% absolute alcohol, followed by infiltration with molten paraffin wax at 60 °C. Subsequent to this preparation, staining was carried out using hematoxylin and eosin dye. The prepared slides were then poised for histopathological examination, and photomicrographs were captured using a microscope under a 40× magnification lens.

### 2.12. Statistical Analysis

Statistical analysis was conducted using GraphPad Prism 5 (GraphPad Software Inc., La Jolla, CA, USA). One-way ANOVA and Two-way ANOVA were utilized to determine significant differences between the groups, considering probability values of (*p* < 0.05) as thresholds for significance. The presentation of graphical data included mean values and standard deviations (SD).

## 3. Results

### 3.1. Effect on Body Weight

**Figure 2:**
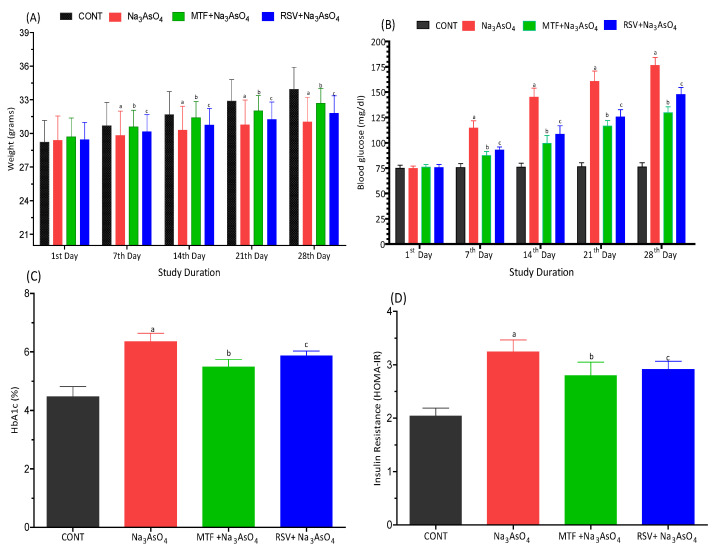
Effect of arsenic exposure and treatment on (**A**) body weight, (**B**) glucose level, (**C**) HbA1c and (**D**) HOMA-IR. “a” Significant changes when comparing the arsenic-exposed group with the control group. “b” Significant changes when comparing the arsenic-exposed group with the standard-treated group and “c” Significant changes when comparing the arsenic-exposed group with the resveratrol-treated group.

The initial body weights of all mice were recorded at the beginning of the study. The average initial body weights for mice in the CONT, Na_3_AsO_4_, MTF + Na_3_AsO_4_, and RSV + Na_3_AsO_4_ groups were 29.51 g, 29.38 g, 29.69 g, and 29.43 g, respectively. Throughout the study, all mice were provided with standard mice chow and water as needed on a daily basis. Subsequently, body weights of all mice were measured prior to feeding on the 7th, 14th, 21st, and 28th days. No significant weight gain was observed across any group on the 7th day of the study. However, as the study progressed, notable differences in weight gain emerged. Particularly, a significant variation (*p* < 0.001) in weight gain was noted within the group exposed to sodium arsenate in comparison to the control group. The presence of sodium arsenate seemed to hinder the growth of mice compared to the control group. The body weight gain in the sodium arsenate-exposed mice was significantly reduced (approximately 10% less) compared to the control group by the 28th day (*p* < 0.001). The average weight gain in the CONT group was 33.93 g (an increase of 16% from the initial weight), while in the sodium arsenate-exposed group it was 31.01 g (a 5.5% increase from the initial weight). Furthermore, the weight gain within the group receiving standard treatment (32.69 g) or RSV treatment (32.54 g) exhibited a pattern comparable to that of the control group. A graphical representation of the changes in body weight among the treated groups is provided in Figure 2A.

### 3.2. Effect on Glycemic Index Biomarkers

We initially assessed the glucose levels of all mice before the study commenced. Subsequently, glucose levels were measured in a fasting state on the 7th, 14th, 21st, and 28th days. By the 7th day of the study, an increase in glucose levels was observed in the group exposed to arsenic. A significant hyperglycemic effect was noted in the sodium arsenate-exposed group when compared to both the standard-treated group and the RSV-treated group. This effect was also evident when compared to the control group (*p* < 0.001), as depicted in Figure 2B. The values of HbA1c and HOMA-IR also showed an increase within the sodium arsenate-exposed group, as opposed to the standard-treated group or the RSV-treated group (*p* < 0.05). These findings suggested that standard treatment or RSV treatment contributed to the improvement of blood glucose levels. These results are illustrated in Figure 2C,D.

### 3.3. Effect on Carbohydrate Metabolism Biomarkers

**Figure 3:**
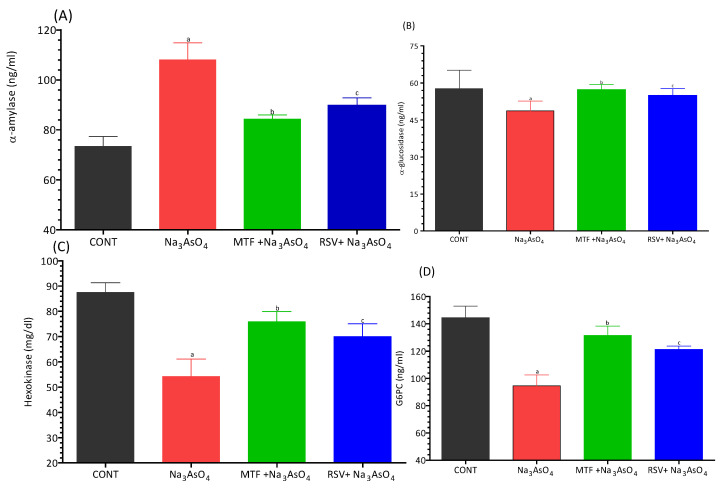
Effect of arsenic exposure and treatment on (**A**) α-amylase, (**B**) α-glucosidase, (**C**) hexokinase and (**D**) G6PC. Level of significant difference was determined by one way ANOVA followed by Bonferroni’s post-test. “a” Significant changes when comparing the arsenic-exposed group with the control group. “b” Significant changes when comparing the arsenic-exposed group with the standard-treated group and “c” Significant changes when comparing the arsenic-exposed group with the resveratrol-treated group.

We examined the impact of sodium arsenate on the activity of carbohydrate metabolic enzymes, considering the group of mice that received standard treatment or RSV treatment to counteract the effects of arsenic. We observed a decrease in the levels of α-glucosidase (mean = 48.67 ng/mL ± 3.977), hexokinase (mean = 54.17 ng/mL ± 7.028), and G6PS (mean = 94.50 ng/mL ± 8.044) within the sodium arsenate-exposed group. These levels were significantly lower than those observed in the control group (*p* < 0.05). Both the standard drug and RSV treated mice had improved levels of these enzymes, like α-glucosidase, hexokinase and G6PS, at the arsenic site. Treated group showed comparable results and there was no significant difference in the levels of these metabolic enzymes between the groups that received standard treatment or RSV treatment. Notably, the serum α-amylase level exhibited a slight increase (mean = 84.64 ng/mL ± 2.330) in the mice exposed to sodium arsenate when compared with the normal or treated groups. These findings are presented in Figure 3A–D.

### 3.4. Effect on Biomarkers of Oxidative Stress and Lipid Peroxidation

**Figure 4:**
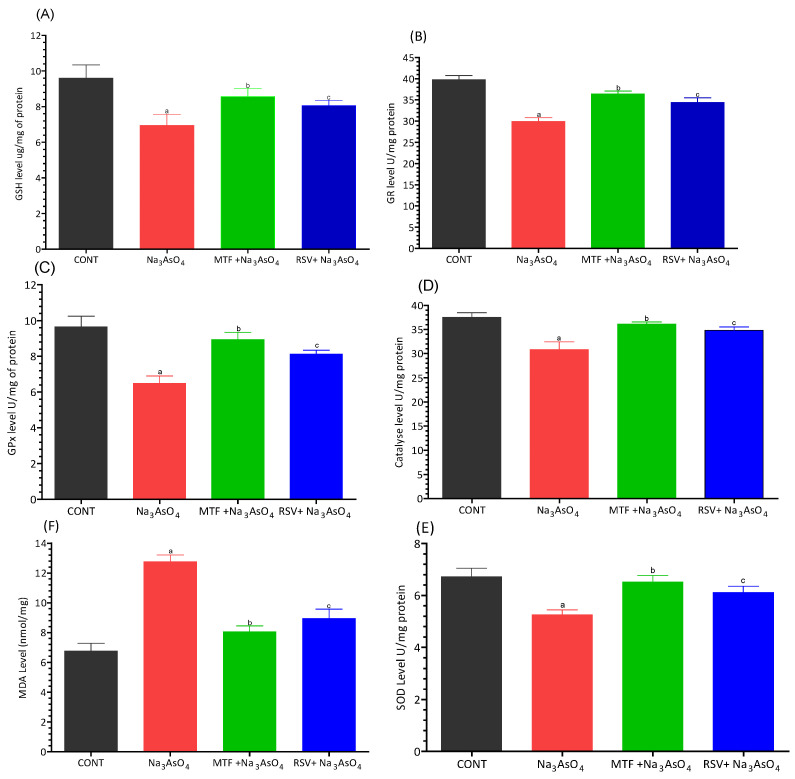
Effect of arsenic exposure and treatment on (**A**) GSH, (**B**) GR, (**C**) GPx, (**D**) Catalase, (**E**) SOD and (**F**) MDA. Level of significant difference was determined by one way ANOVA followed by Bonferroni’s post-test. “a” significant change when comparing the arsenic exposed group with the control group. “b” Significant change when comparing the arsenic exposed group with the standard treated group and “c” Significant change when comparing the arsenic exposed group with the resveratrol treated group.

The results of our study following sodium arsenate intoxication revealed a significant reduction in GSH, GR, GPx, Catalase, and SOD levels (*p* < 0.05) in the mice exposed to sodium arsenate. In contrast, the level of MDA exhibited a significant increase (*p* < 0.05). The levels of GPx, Catalase, and SOD were significantly improved in mice treated either with standard drug or group received RSV along with exposure to arsenic. The ameliorating effect of RSV was comparable with the standard drug and no significant deviation was reported from the control group. Both groups demonstrated improved GPx, Catalase, and SOD levels when compared with the control group. These observations are depicted in Figure 4A–F.

### 3.5. Effect on Inflammatory, Kidney and Liver Biomarkers

**Figure 5:**
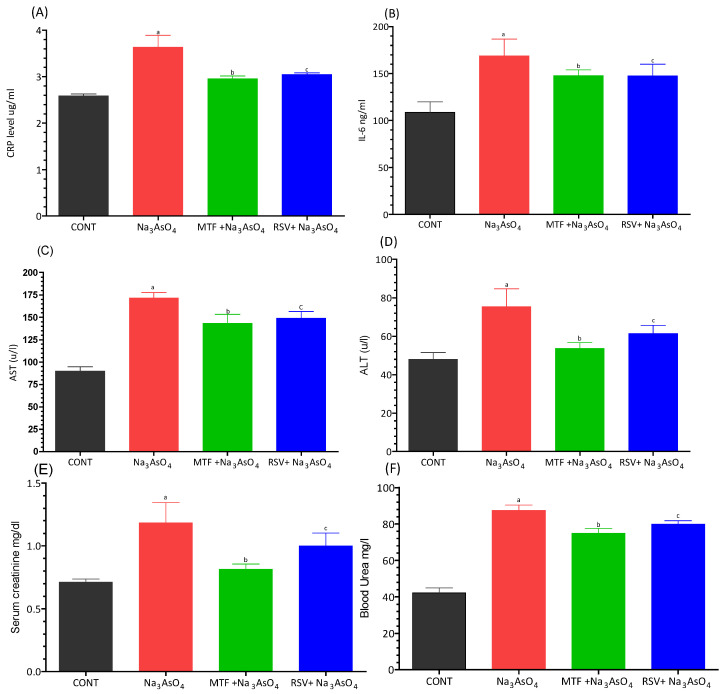
Effect of arsenic exposure and treatment on (**A**) CRP, (**B**) IL-6, (**C**) AST, (**D**) ALT, (**E**) serum creatinine and (**F**) blood urea. Level of significant difference was determined by one way ANOVA followed by Bonferroni’s post-test. “a” Significant changes when comparing the arsenic-exposed group with the control group. “b” Significant changes when comparing the arsenic-exposed group with the standard-treated group and “c” Significant changes when comparing the arsenic-exposed group with the resveratrol-treated group.

The levels of inflammatory biomarkers, i.e., C-reactive protein and IL-6, exhibited a significant increase (*p* < 0.05) in the group exposed to sodium arsenate (Figure 5A,B). Conversely, the group that received MF or RSV in addition to sodium arsenate demonstrated a notable reduction in the levels of these inflammatory biomarkers compared to the arsenic-exposed group. Similarly, the liver biomarkers, i.e., ALT and AST, as well as the kidney biomarkers, serum creatinine and blood urea, displayed a significant increase (*p* < 0.05) in the arsenic-exposed group when compared to the standard-treated and RSV-received groups (Figure 5C–F).

### 3.6. Estimation of Arsenic by ICP-MS

**Figure 6:**
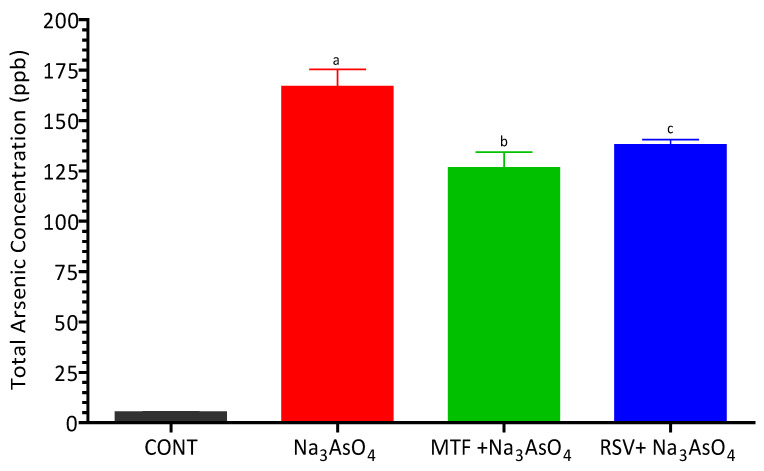
Detection of total arsenic content in serum of arsenic-exposed mice. Level of significant difference was determined by one way ANOVA followed by Bonferroni’s post-test. “a” Significant changes when comparing the arsenic-exposed group with the control group. “b” Significant changes when comparing the arsenic-exposed group with the standard-treated group and “c” Significant changes when comparing the arsenic-exposed group with the resveratrol-treated group.

A noteworthy increase (*p* < 0.05) in total arsenic content was observed in the arsenic-exposed group, with a mean value of 166.80 ± 8.52 ppb, in comparison to the control group, which exhibited a mean value of 5.26 ± 0.10 ppb (Figure 6). This stark contrast underscores the significant arsenic exposure experienced by the arsenic-exposed group. Moreover, the concentrations of arsenic showed a substantial elevation in both the MF group (126.50 ± 7.90 ppb) and the RSV group (137.90 ± 2.52 ppb) when contrasted with the control group (*p* < 0.05). This finding implies that, even in the groups receiving the potential remedies (MF and RSV), the presence of elevated arsenic concentrations was notable. A clear visualization of these reported concentrations is provided in Figure 6, depicting the extent of arsenic exposure across the different experimental groups.

### 3.7. Gene Expression

**Figure 7:**
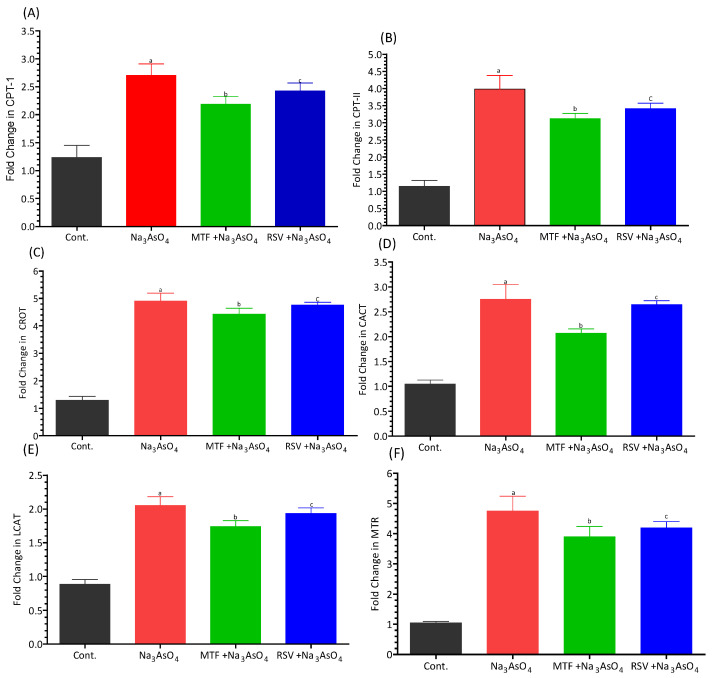
Effect of arsenic exposure and treatment on (**A**) CPT I, (**B**) CPT II, (**C**) LCAT, (**D**) CROT, (**E**) CACT, and (**F**) MTR. Level of significant difference was determined by one way ANOVA followed by Bonferroni’s post-test. “a” Significant changes when comparing the arsenic-exposed group with the control group. “b” Significant changes when comparing the arsenic-exposed group with the standard-treated group and “c” Significant changes when comparing the arsenic-exposed group with the resveratrol-treated group.

**Figure 8:**
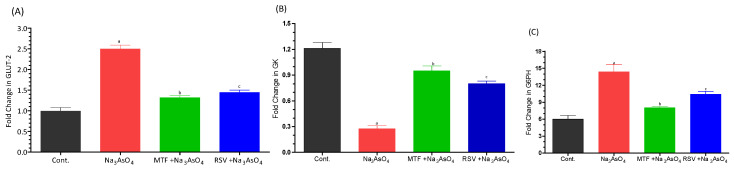
Effect of arsenic exposure and treatment on (**A**) GLUT-2, (**B**) GK and (**C**) G6PC. Level of significant difference was determined by one way ANOVA followed by Bonferroni’s post-test. “a” Significant changes when comparing the arsenic-exposed group with the control group. “b” Significant changes when comparing the arsenic-exposed group with the standard-treated group and “c” Significant changes when comparing the arsenic-exposed group with the resveratrol-treated group.

We have documented a notable upregulation in the gene expression of key enzymes involved in lipid metabolism and amino acid metabolism, including carnitine palmitoyl-transferase-I (CPT-I), carnitine palmitoyl-transferase-II (CPT-II), lecithin–cholesterol acyltransferase (LCAT), carnitine O-octanoyl-transferase (CROT), mitochondrial carnitine/acylcarnitine carrier protein (CACT), and 5-methyltetrahydrofolate-homocysteine methyltransferase (MTR), in the liver of mice exposed to sodium arsenate. These genes play pivotal roles in facilitating various aspects of lipid and amino acid metabolism. Importantly, this upregulation exhibited statistical significance when compared to the control group, as well as when compared to both the standard-treated group and the RSV-treated group (*p* < 0.05). In contrast, the co-treatment group, which received RSV in addition to sodium arsenate, demonstrated a substantial decrease in the expression levels of these genes (*p* < 0.05), indicating a potential mitigating effect of RSV on the arsenic-induced upregulation of these lipid and amino acid metabolism-related genes. Furthermore, we observed an upregulation in the gene expression of Glucose transporter-2 (GLUT-2) and glucose-6-phosphatase (G6PC), along with a downregulation in the expression of Glucokinase (GK), all of which are involved in carbohydrate metabolism. The gene expression of GLUT-2 and G6PC demonstrated significant increases in relation to the control group (*p* < 0.05), indicating potential disruptions in carbohydrate metabolism due to arsenic exposure. These intricate patterns of gene expression are visually represented in Figure 7A–F for the lipid and amino acid metabolism-related genes, and in Figure 8A–C for the carbohydrate metabolism-related genes.

### 3.8. Analysis of Amino Acid

**Figure 9:**
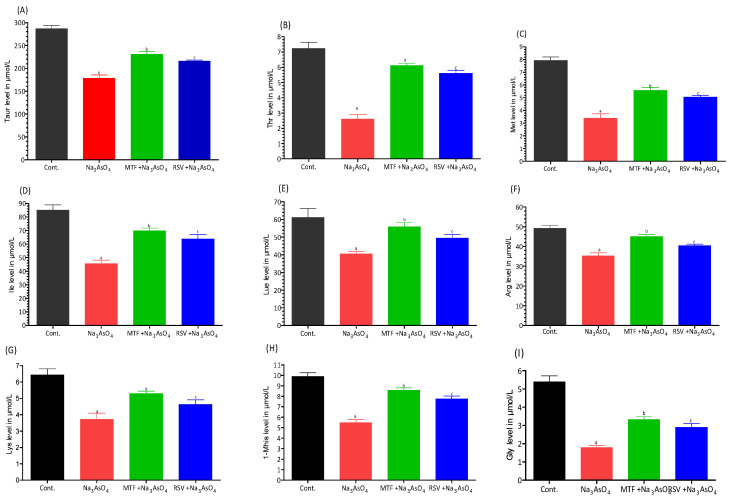
Effects of arsenic exposure and treatment on different amino acid levels were determined using an amino acid analyzer. The amino acids analyzed were (**A**) Taurine, (**B**) Threonine, (**C**) Methionine, (**D**) Isoleucine, (**E**) Leucine, (**F**) Arginine, (**G**) Lysine, (**H**) 1-Methylhistidine, and (**I**) Glycine. The level of significant differences was determined by a one-way ANOVA followed by Bonferroni’s post-test. “a” Significant changes when comparing the arsenic-exposed group with the control group. “b” Significant changes when comparing the arsenic-exposed group with the standard-treated group and “c” Significant changes when comparing the arsenic-exposed group with the resveratrol-treated group.

We identified 12 amino acids in the plasma of mice using the Amino Acid Analyzer. Notably, among these amino acids, nine—Taurine, Threonine, Methionine, Isoleucine, Leucine, Arginine, Lysine, L-Methionine sulfoxide, and Glycine—showed significant reductions in the mice exposed to arsenic (Figure 9).

### 3.9. Metabolomics Analysis

#### 3.9.1. Analysis of Metabolomes by MS/MS

We acquired data using both positive (ESI+) and negative (ESI−) electrospray ionization modes, while maintaining the capillary voltage at 4.7 kV. Data collection was conducted within the *m*/*z* range of 50 to 2000. Multiple peaks were selected for fragmentation through collision-induced dissociation (CID), employing energy levels spanning from 20 to 30. Peak selection was solely based on the accurate molecular mass of the compound of interest in Collision-Induced Dissociation (CID), along with the obtained fragmentation ion peaks. A literature review further confirmed the precursor ion peak and its associated daughter ion peaks. This comprehensive approach allowed us to identify numerous metabolites encompassing lipid metabolites, amino acids, and arsenic metabolites.

#### 3.9.2. Lipid Metabolomes Based on Peak Analysis from Database

A diverse array of compounds spanning different lipid classes, including fatty acyls, glycerolipids, glycerophospholipids, sphingolipids, and sterol lipids, were detected in both the arsenic-exposed group and the group treated with resveratrol following arsenic exposure. These compounds were identified by accurately matching their masses against online databases, such as LIPID MAPS (http://www.lipidmaps.org/, accessed on 12 April 2023). The identified lipid classes encompassed fatty acids/esters (FA), tri(acyl|alkyl)glycerols (TG), glycero-phosphocholines (PC), glycerophosphates (PA), glycero-phosphoserines (PS), glycero-phosphoethanolamines (PE), glycero-phosphoglycerols (PG), glycero-phosphoinositols (PI), sphingolipids, like Hexosyl ceramides (HexCer) and Ceramide phosphates (CerP), and sterol lipids, such as Sterols and cholesterol esters (ST and CE).

**Figure 10:**
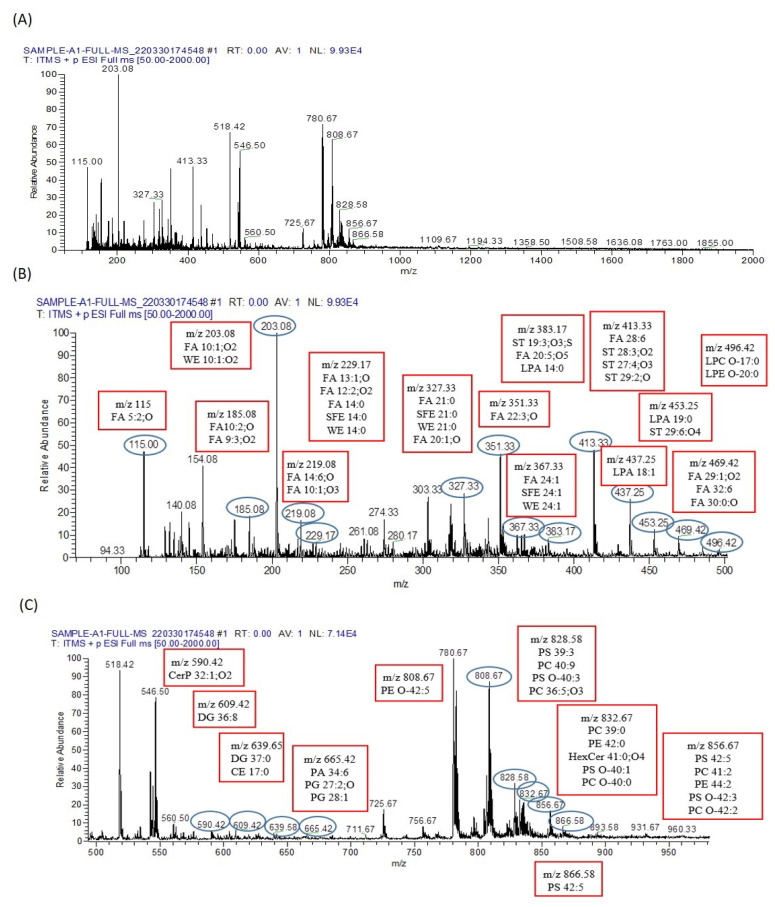
Metabolomic analysis of arsenic-exposed mice serum. (**A**) The ESI+-MS/MS spectrum covers the *m*/*z* range from 0 to 2000. To facilitate detailed analysis, this broad range is divided into two segments, (**B**,**C**), each processed to extract crucial peak pair information. The distinct peak pairs obtained from differential labeling of metabolites in the sample analysis are apparent in both segments. (**B**) The segmented ESI+-MS/MS spectrum, focusing on the *m*/*z* range from 0 to 500, is labeled with the identified lipid metabolomes. For instance, LPC O-17;0 is detected at *m*/*z* 496.42, providing valuable insights into the lipidomic composition of the sample. (**C**) Continuing the analysis, the segmented ESI+-MS/MS spectrum delves into the *m*/*z* range from 500 to 960. This segment is also labeled with identified lipid metabolomes, such as HexCer 41;0;O4 at *m*/*z* 832.67 and PC O-42;2 at *m*/*z* 856.67. These identified lipid metabolites contribute to a comprehensive understanding of the metabolic landscape in the serum of arsenic-exposed mice.

In the serum of arsenic-exposed mice, 57 metabolites were identified using the LIPID MAP database in full MS/MS ESI+ mode, as illustrated in Figure 10.

**Figure 11:**
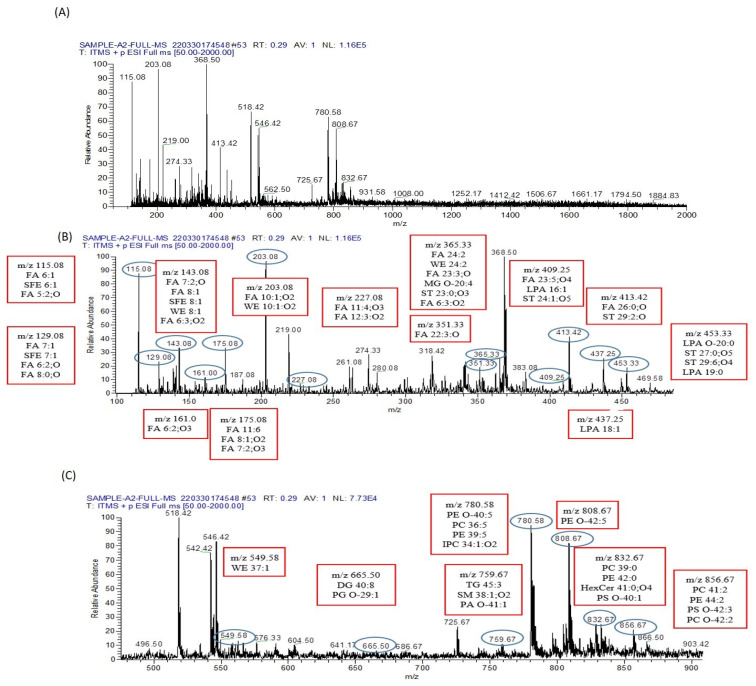
Metabolomic analysis of resveratrol-plus-arsenic-exposed mice serum. (**A**) The ESI+-MS/MS spectrum covers the *m*/*z* range from 0 to 2000, providing a comprehensive view of the metabolites present. To enhance analysis, this wide range is divided into two distinct segments, (**B**,**C**), each meticulously processed to extract peak pair information. These peak pairs correspond to differentially labeled metabolites within the sample. (**B**) The segmented ESI+-MS/MS spectrum, focused on the *m*/*z* range from 100 to 500, is labeled with identified lipid metabolomes. Notably, LPA O-20;0 is observed at *m*/*z* 453.33, contributing valuable insights into the composition of lipid metabolites within the sample. (**C**) Continuing the investigation, the segmented ESI+-MS/MS spectrum explores the *m*/*z* range from 500 to 900. This segment is also labeled with identified lipid metabolomes, further enhancing our understanding of the lipidomic profile within the serum of mice exposed to both resveratrol and arsenic. These analyses collectively provide a comprehensive picture of the metabolic alterations induced by the combined exposure to resveratrol and arsenic in the mice serum.

**Figure 12:**
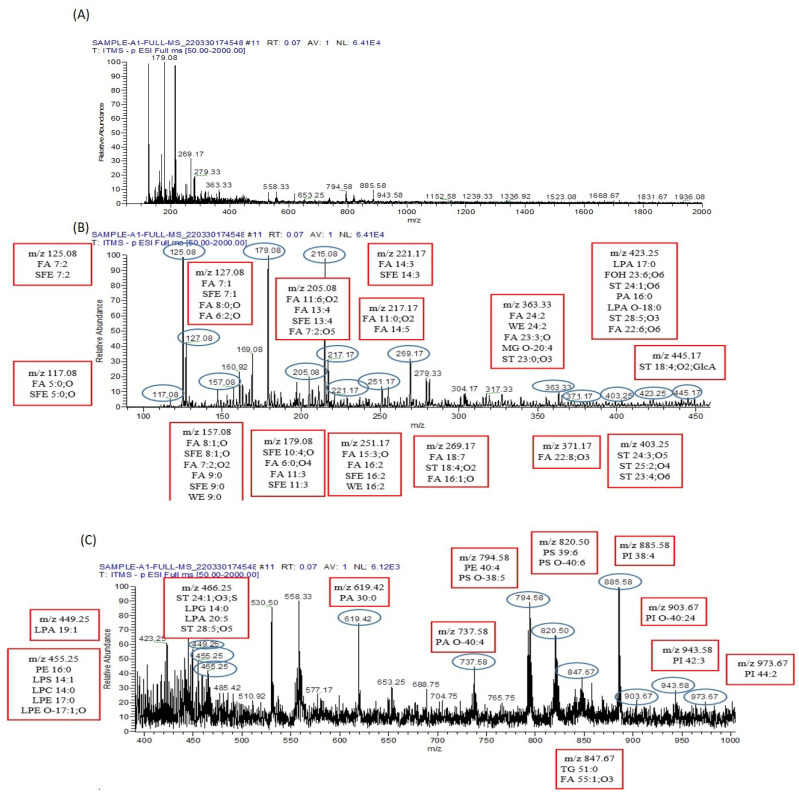
Metabolomic analysis of arsenic-exposed mice serum. (**A**) The ESI−-MS/MS spectrum spans the *m*/*z* range from 0 to 2000, capturing a comprehensive array of metabolites present in the sample. To facilitate a detailed investigation, this expansive range is segmented into two sections, (**B**,**C**), each meticulously processed to extract peak pair information. These peak pairs correspond to differentially labeled metabolites, furnishing essential insights into the sample’s composition. (**B**) The segmented ESI−-MS/MS spectrum, covering the *m*/*z* range from 0 to 450, is labeled with identified lipid metabolomes. This segment enables a focused analysis of lipidomic components within the sample, shedding light on their potential alterations induced by arsenic exposure. (**C**) Moving forward, the segmented ESI+-MS/MS spectrum explores the *m*/*z* range from 400 to 960. This segment is similarly labeled with identified lipid metabolomes, enriching our understanding of the lipidomic profile within the serum of mice exposed to arsenic. These analyses collectively offer a comprehensive view of the metabolic changes brought about by arsenic exposure in the mice serum.

**Figure 13:**
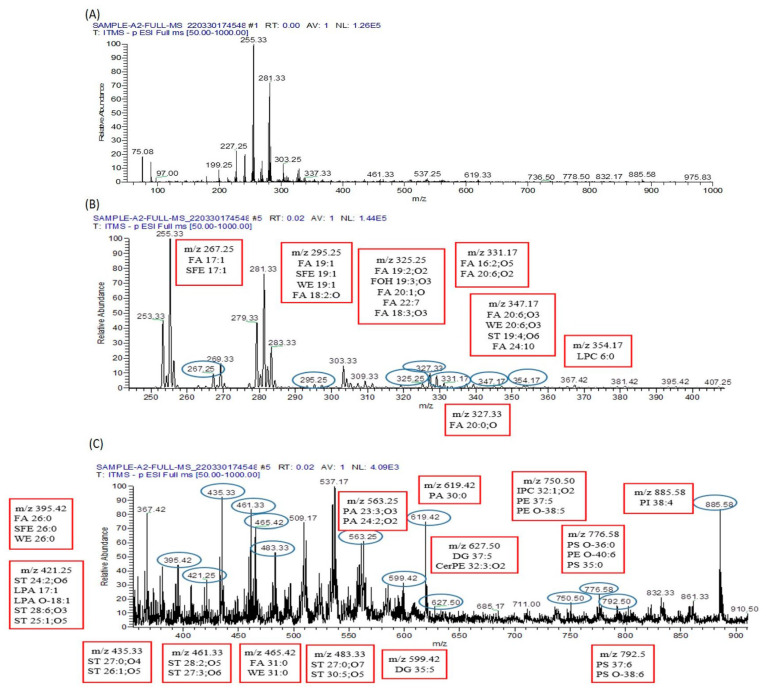
Metabolomic analysis of resveratrol-plus-arsenic-exposed mice serum. (**A**) The ESI−-MS/MS spectrum spans the *m*/*z* range from 0 to 1000, encompassing a broad array of metabolites present in the sample. To facilitate a comprehensive investigation, this range is divided into two distinct segments, (**B**,**C**). Each segment is meticulously processed to extract peak pair information, where every peak pair corresponds to differentially labeled metabolites, enriching our understanding of the sample’s composition. (**B**) Within the segmented ESI−-MS/MS spectrum, focusing on the *m*/*z* range from 0 to 400, identified lipid metabolomes are labeled. This targeted analysis zooms in on the lipidomic components within the sample, offering insights into their potential alterations resulting from the dual exposure to resveratrol and arsenic. (**C**) Transitioning to the segmented ESI+-MS/MS spectrum, spanning the *m*/*z* range from 400 to 900, identified lipid metabolomes are once again labeled. This segment further unravels the lipidomic profile within the serum of mice exposed to both resveratrol and arsenic. The combined analyses provide a comprehensive overview of the metabolic changes induced by the simultaneous exposure to resveratrol and arsenic in the mice serum.

Conversely, 56 metabolites were identified in the serum of arsenic-exposed mice treated with resveratrol, as shown in Figure 11. Interestingly, only eleven metabolites were found to be common between the arsenic-exposed and arsenic-exposed treated groups. These shared metabolites include PC 41:2, PE 44:2, PS O-42:3, PC O-42:2, PC 39:0, PE 42:0, HexCer 41:0;O4, PS O-40:1, PC O-40:0, PE O-42:5, ST 29:6;O4, LPA 18:1, FA 22:3;O, and FA 5:2;O. In full MS/MS ESI− mode, 75 metabolites were identified in the serum of arsenic-exposed mice (Figure 12), while 50 metabolites were identified in the serum of arsenic-exposed mice treated with resveratrol (Figure 13). Notably, the metabolites identified in ESI− mode were entirely distinct, with the exception of two metabolites—PA 30:0 and ST 28:5;O5.

**Figure 14:**
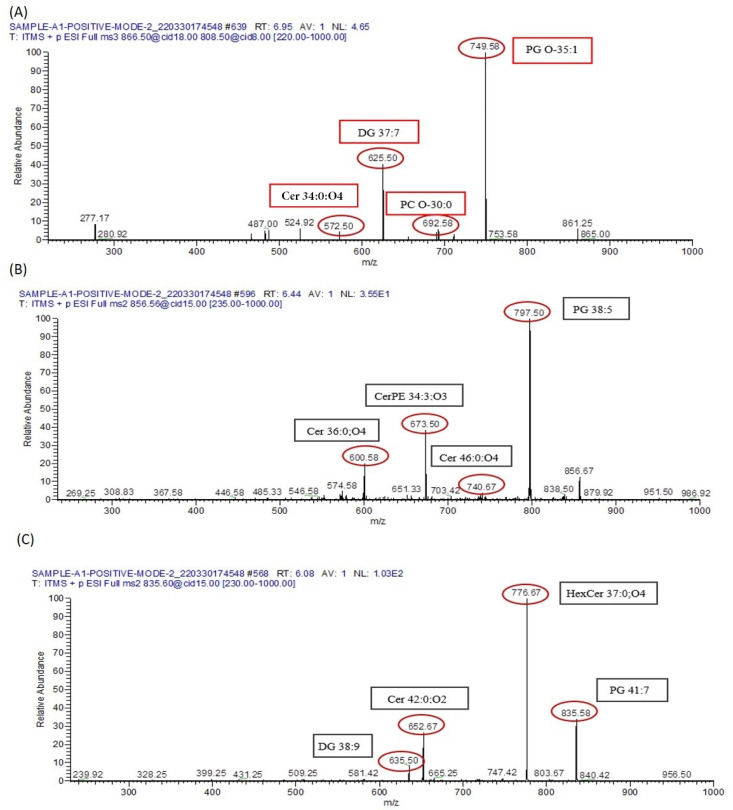
Disruptive lipid metabolomes identified via MS/MS. (**A**) The MS/MS spectrum at *m*/*z* 835.60, captured within the *m*/*z* range of 220 to 1000, exposes lipid metabolomes instrumental in the disturbance of metabolic pathways. (**B**) Focusing on the MS/MS spectrum obtained at *m*/*z* 856, spanning the *m*/*z* range of 235 to 1000, further illuminates specific lipid metabolomes that play a role in metabolic disruption. (**C**) Expanding the investigation to the MS/MS spectrum at *m*/*z* 866.5, covering the *m*/*z* range from 230 to 1000, provides additional insights into disruptive lipid metabolomes.

Due to the extensive presence of lipid isomers, the database search unveiled multiple potential compounds sharing the same *m*/*z* value for various lipids. Consequently, identification solely based on precise mass is insufficient for a conclusive determination. For instance, in the positive ESI mode MS/MS spectrum captured at *m*/*z* 866.5, targeted lipid metabolomes such as PG O-35:1 (*m*/*z* 749.58), PC O-30:0 (*m*/*z* 692.58), DG 37:7 (*m*/*z* 625.5), and Cer 34:0;O4 (*m*/*z* 572.5) were detected in the case of arsenic-exposed mice. Similarly, in the positive ESI mode MS/MS spectrum taken at *m*/*z* 856.67, PG 38:5 (*m*/*z* 797.5), Cer 46:0;O4 (*m*/*z* 740.67), CerPE 34:3;O3 (*m*/*z* 673.5), and Cer 36:0;O4 (*m*/*z* 600.58) were identified in the serum samples of arsenic-exposed mice. Furthermore, the MS/MS spectrum obtained at *m*/*z* 866.5 in positive ESI mode revealed the presence of PG 41:7 (*m*/*z* 835.58), HexCer 37:0;O4 (*m*/*z* 776.67), Cer 42:0;O2 (*m*/*z* 652.67), and DG 38:9 (*m*/*z* 635.5) in the same group, as represented in Figure 14. These detected lipid metabolomes primarily contribute to the disruption of several crucial metabolic pathways. For example, Diacylglycerol induces insulin resistance by activating PKC-ε and reducing IRS-2 tyrosine phosphorylation. Ceramide is significant in sphingolipid metabolism and is linked to insulin resistance and metabolic dysregulation. Glycero-phosphocholines contribute to insulin resistance (IR) and glucose intolerance.

#### 3.9.3. Identification of Metabolomes with Molecular Ion Fragmentation Method

A range of compounds was identified using the ion fragmentation method, as outlined in Table 3 and Table 4. These compounds encompass Sphinganine, Phyto-sphingosine, Lysophosphatidyl-choline, Carnitine, Arginine, Dimethylarginine, Isoleucine, Valine, Threonine, and arsenic metabolites.

#### 3.9.4. Lipid Metabolomes

##### Sphinganine

**Figure 15:**
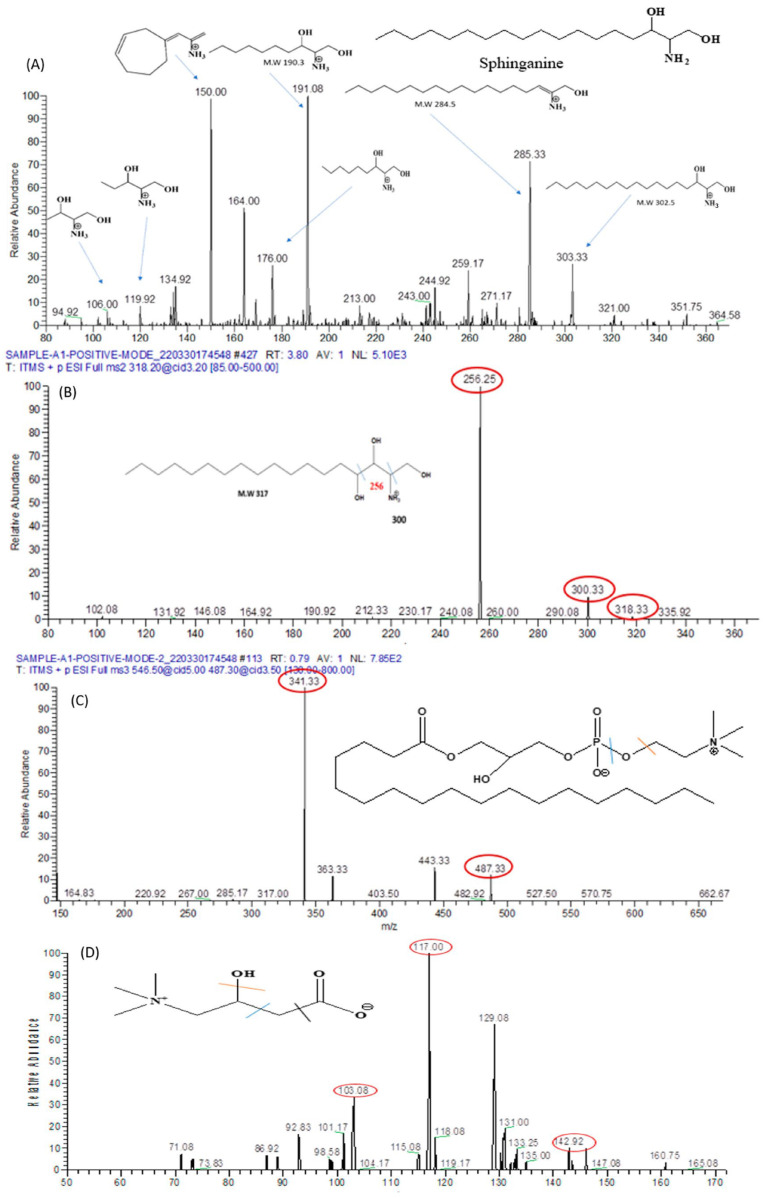
Detected lipid metabolomes via MS/MS analysis in treated arsenic-exposed mice. (**A**) The MS/MS spectrum in ESI+ mode of Sphinganine at *m*/*z* 303.33 is meticulously presented. The spectrum not only highlights the presence of Sphinganine but also provides a detailed view of its fragment peaks. (**B**) Building on this, the MS/MS spectrum in ESI+ mode of phyto-sphingosine at *m*/*z* 318.20 is elucidated. This spectrum offers insights into the characteristics of phyto-sphingosine, shedding light on its structural features and potential modifications. (**C**) The investigation extends to the MS/MS spectrum in ESI+ mode of lysophosphatidyl-choline at *m*/*z* 546.50. (**D**) The analysis also includes the MS/MS spectrum in ESI− mode of Carnitine at *m*/*z* 160.90.

Sphinganine is an active participant in the sphingolipid metabolism pathway. With a molecular weight of 302, its presence is evident in the MS/MS analysis’s positive ion mode, as illustrated in Figure 15A. Distinctive fragmentation peaks were discerned at 285 *m*/*z*, arising from the elimination of a water molecule. Additionally, peaks at 190.3 *m*/*z*, 176.3 *m*/*z*, 119.92 *m*/*z*, and 106 *m*/*z* emerged due to the detachment of alkyl chains encompassing 8, 9, 13, and 14 carbons, respectively. The apparent convergence of the peak at 190.3 *m*/*z* with the peak at 191.08 *m*/*z* was ascribed to an isotope effect.

##### Phyto-Sphinganine

Phyto-sphingosine serves as a fundamental constituent within sphingolipids. With a molecular weight of 317, it displays a characteristic peak at 318 *m*/*z* in the MS/MS spectrum under ESI positive ion mode, as depicted in Figure 15B. This identification is corroborated by the occurrence of fragment peaks at 300 *m*/*z*, originating from the elimination of 2-hydroxyethyl amine, as well as at 256 *m*/*z* resulting from the loss of a water molecule.

##### Lysophosphatidyl-Choline

Lysophosphatidyl-choline (LysoPC) possesses a molecular mass of 523 and manifests a characteristic peak at 546 *m*/*z* in the MS/MS spectrum under positive ion mode, as illustrated in Figure 15C. This peak results from the incorporation of a sodium ion. In succession, a fragmentation peak emerges at *m*/*z* 487, denoting the elimination of a trimethylammonium ion. Subsequently, this latter peak undergoes further dissociation, yielding two additional peaks located at *m*/*z* 341 and 443.

#### 3.9.5. L-Carnitine

Carnitine possesses a molecular weight of 162. The peak at 160.75 in the MS/MS spectrum confirms its presence in the serum sample, particularly evident in the negative ion mode, as depicted in Figure 15D. This is further validated by fragmentation peaks observed at 142.9 *m*/*z* after the elimination of a water molecule, 117 *m*/*z* subsequent to the detachment of the carboxylic group, and 103.08 *m*/*z* following the removal of ethanoic acid.

### 3.10. Identified Amino Acids

#### 3.10.1. Arginine

**Figure 16:**
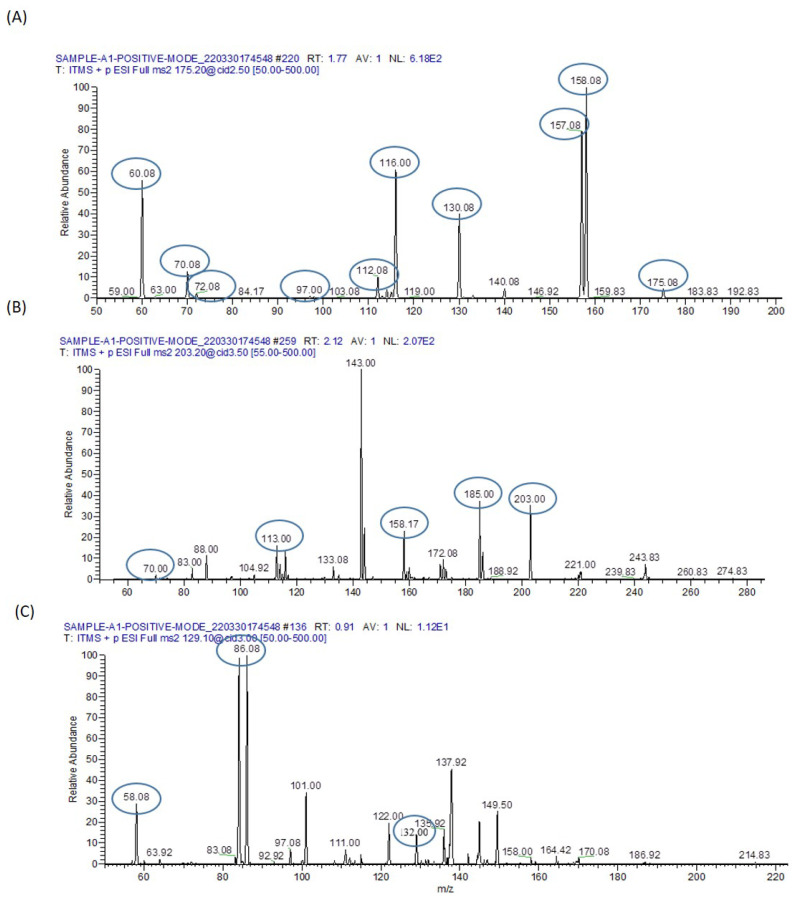
MS/MS spectrum of identified amino acids in treated samples of arsenic-exposed mice. (**A**) The MS/MS spectrum in ESI+ mode of Arginine at *m*/*z* 175.20 is meticulously showcased. The major fragment peaks at *m*/*z* 116.00 and *m*/*z* 60.08 arise due to the elimination of the guanidine group (HN = C(NH_2_)_2_) and C_5_H_9_NO_2_, respectively. Additionally, three minor fragment peaks at *m*/*z* 158.08, *m*/*z* 157.08, and *m*/*z* 130.08 emerge from the loss of NH_3_, H_2_O, and NH_3_^+^ CO. (**B**) Expanding this exploration, the MS/MS spectrum in ESI+ mode of Dimethylarginine at *m*/*z* 203.20 is elucidated. Notably, fragment ions at *m*/*z* 185, 172.08, 158.17, 113, 88, and 70 are detected. The presence of the peak at *m*/*z* 70 is attributed to dimethyl-carbodiimidium and/or cyano-dimethylammonium, while the peak at *m*/*z* 88 originates from dimethylated guanidinium. (**C**) The analysis extends to the MS/MS spectrum in ESI+ mode of isoleucine. This spectrum focuses on the features of isoleucine and its fragmentation behavior. Notably, the characteristic peak at *m*/*z* 132.0 is highlighted, and fragment ions C_5_H_12_N^+^ at *m*/*z* 86.08 and C_3_H_8_N^+^ at *m*/*z* 58.8 are observed.

Arginine possesses a molecular mass of 174.20 and exhibits a distinctive peak at 175.08, as depicted in Figure 16A. Upon the dissociation of protonated arginine, two major fragment ions emerge at *m*/*z* 116.00 and *m*/*z* 60.08, stemming from the elimination of HN = C(NH_2_)_2_ (guanidine group, CH5N3) and C_5_H_9_NO_2_. The fragment at *m*/*z* 116.00 further yields a predominant fragment ion at *m*/*z* 70.08 via the removal of H_2_O + CO. Additionally, three minor fragments arise at *m*/*z* 158.08, *m*/*z* 157.08, and *m*/*z* 130.08 due to the loss of NH_3_, H_2_O, and NH_3_ + CO. Moreover, the concurrent elimination of NH_3_ + CO_2_ from the fragment ion at *m*/*z* 158.08 results in the formation of a fragment ion at *m*/*z* 97.00. Similarly, the detachment of H_2_O + CO from the fragment ion at *m*/*z* 158.08 generates a fragment ion at *m*/*z* 112.08. Notably, the high collision energy-induced separation of the guanidine group from the fragment ion at *m*/*z* 130.08 gives rise to a minor fragment ion at *m*/*z* 72.08 by eliminating the guanidine group from the parent ion.

#### 3.10.2. Dimethylarginine

Dimethylarginine possesses a molecular mass of 202.25 and displays a precursor molecular ion peak at *m*/*z* 203, accompanied by fragment ions at *m*/*z* 185, 172.08, 158.17, 113, 88, and 70, as depicted in Figure 16B. The peak at *m*/*z* 70 is most likely ascribed to dimethyl-carbodiimidium and/or cyano-dimethylammonium, while the peak at *m*/*z* 88 originates from dimethylated guanidinium. Both of these fragment ions serve as indicative markers of dimethylarginine residues.

#### 3.10.3. Isoleucine

Isoleucine possesses a molecular mass of 132.1 with the ion formula (C6H14NO2+), and it presents a distinctive peak at *m*/*z* 132.0. It gives rise to a fragment ion C5H12N+ at *m*/*z* 86.08 and C3H8N+ at *m*/*z* 58.8, as illustrated in Figure 16C.

#### 3.10.4. Miscellaneous Metabolites

**Figure 17:**
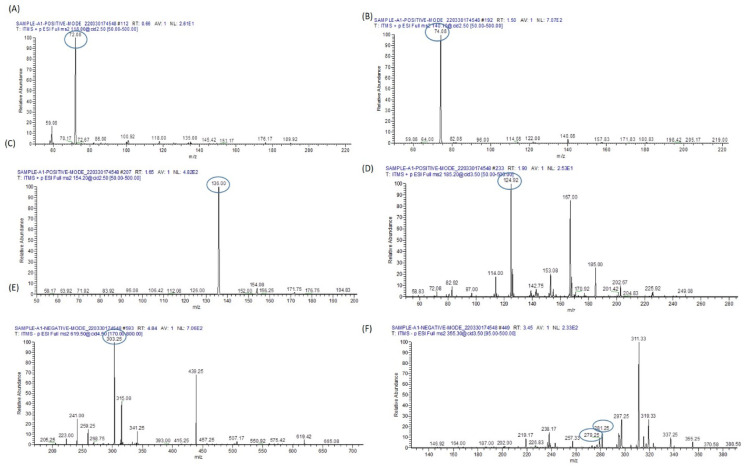
Characteristic peaks of identified metabolites in treated samples of arsenic-exposed mice. (**A**) The representation of the characteristic peak of Valine at *m*/*z* 72.08 is presented in detail. (**B**) The representation of the characteristic peak of Threonine at *m*/*z* 74.08 is meticulously depicted. (**C**) The depiction extends to the representation of the characteristic peak of Homocysteine at *m*/*z* 136. (**D**) The characteristic peak of Taurine at *m*/*z* 124.92. (**E**) The characteristic peak of Arachidonic acid at *m*/*z* 303.25 is presented. (**F**) The characteristic peak of Linoleic acid at *m*/*z* 279.25 and Gamma-Linoleic acid at *m*/*z* 281.25.

In Figure 17, we have identified the characteristic peak of Valine at *m*/*z* 72.08, Threonine at *m*/*z* 74.08, Homocysteine at *m*/*z* 136, Taurine at *m*/*z* 124.92, Linoleic acid at *m*/*z* 279.25, Gamma-linolenic acid at *m*/*z* 281.25, and Arachidonic acid at *m*/*z* 303.25 in the serum of mice exposed to sodium arsenate.

### 3.11. Inorganic Arsenic and Its Metabolites

**Figure 18:**
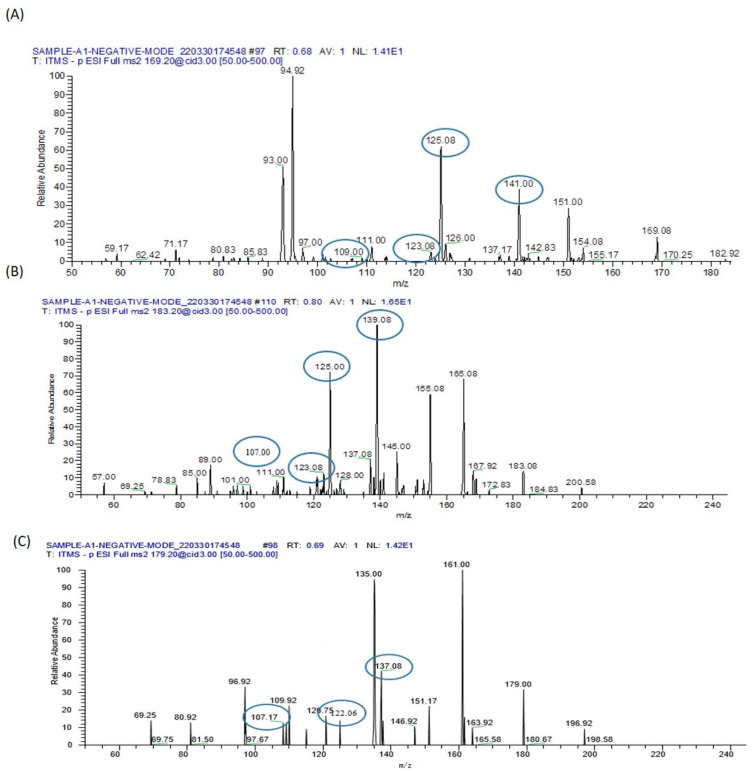
MS/MS Spectrum of various arsenic metabolites. (**A**) The MS/MS spectrum illustrates the characteristic peak of the Arsenate molecular ion at *m*/*z* 141. Additionally, the spectrum showcases its fragment ion at *m*/*z* 123, elucidating the breakdown of the molecular structure. Similarly, the Arsenite molecular ion at *m*/*z* 125 is presented, accompanied by its corresponding fragment ion at *m*/*z* 109. (**B**) This spectrum highlights the distinctive peak at *m*/*z* 139, characteristic of this specific metabolite. Accompanying fragment peaks are also revealed, including those at *m*/*z* 125, 123.08, 121, and 107, offering a detailed view of the fragmentation pattern associated with arsenate monomethyl arsenic acid. (**C**) The spectrum further showcases fragment peaks at *m*/*z* 122 and 107, offering a comprehensive visualization of the fragmentation pattern unique to dimethyl-arsinous acid.

As a consequence of metabolic methylation of inorganic arsenic, various pentavalent metabolites and trivalent arsenic intermediates are generated (Table 4). In this context, we have identified arsenate monomethyl arsenic acid (MMAV) and dimethyl-arsinous acid (DMAIII), each accompanied by their distinctive fragment ions at specific *m*/*z* values, as illustrated in Figure 18.

### 3.12. Histopathology

**Figure 19:**
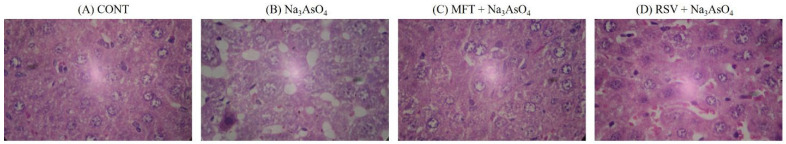
Histopathological Examination of Liver Sections. (**A**) The photomicrograph offers a view of a liver section from mice that received purified water orally for a span of 28 days. Notably, the hepatocytes and cell cords display a healthy appearance, characterized by normal hepatocyte architecture. Additionally, the central vein is intact, surrounded by unaltered blood sinusoids and well-preserved hepatocytes. (**B**) Contrastingly, photomicrograph captures a liver section from mice exposed to arsenic through oral administration for a duration of 28 days. This image exhibits severe histopathological alterations within the liver tissue. Notable changes include vacuolation and distortion of hepatic cell cords, indicating the damaging effects of arsenic exposure on hepatocyte structure. (**C**) The liver section from the same group of mice exposed to arsenic reveals slight inflammation of hepatic cells. This observation is complemented by the presence of slight vacuolation and minimal distortion of cell cords, underscoring the adverse impact of arsenic exposure on liver tissue. (**D**) The photomicrograph portrays liver sections from mice exposed to arsenic and subsequently treated with Resveratrol. The image reflects a moderate improvement in hepatic cell health, evident by the presence of only slight vacuolation and minimal distortion of cell cords. This observation emphasizes the potential of Resveratrol treatment in mitigating the deleterious effects of arsenic on liver tissue.

We identified various histopathological changes, such as distention and hemorrhage in the central and portal veins, loss of normal architecture, degenerated hepatocytes with pyknotic nuclei, vacuolated hepatocytes, and dilation of blood sinusoids, along with an increased number of Kupffer cells in the liver of mice exposed to arsenic. The treatment groups with MFT or RSV showed marked improvement and less damage when concurrently exposed to arsenic. This is illustrated in Figure 19.

## 4. Discussion

Diabetes Mellitus is characterized by uncontrolled hyperglycemia that is associated with persistent oxidative stress and inflammation, which are major pathogenic mechanisms contributing to the continuous increase in fasting plasma glucose (FPG), glycosylated hemoglobin (HbA1c), and homeostatic model assessment of insulin resistance (HOMA-IR) [27]. In our study, a significant hyperglycemic effect was observed upon exposure to sodium arsenate in mice, in comparison to the standard treated group or the RSV treated group, as well as when compared to the control group (*p* < 0.001). The values of HOMA-IR and HbA1c were also elevated in the sodium arsenate exposed group compared to the standard treated group or the RSV treated group, showing the potential of standard treatment or RSV to ameliorate the effect on blood glucose (*p* < 0.05). Mahjabeen et al. (2022) also reported a significant decrease in HOMA-IR and insulin levels due to a reduction in fasting glucose; however, the effect on HbA1c levels was less pronounced [28].

Arsenic has the potential to disrupt the normal carbohydrate metabolic pathway by interfering with various enzymes involved in its metabolism [29,30]. The activity of hexokinase, a crucial enzyme in glycolysis, is inhibited by arsenic exposure. Inorganic As III can also notably inhibit several other enzymes associated with glucose metabolism, such as succinyl Co-A synthase, α-ketoglutarate dehydrogenase, glucose-6-phosphate dehydrogenase, and pyruvate dehydrogenase [31]. We have observed a reduction in the levels of α-glucosidase (mean = 48.67 ng/mL + 3.977), hexokinase (mean = 54.17 mg/mL + 7.028), and G6PS (mean = 94.50 ng/mL + 8.044) in the sodium arsenate exposed group, which were significantly lower compared to the control group (*p* < 0.05). The group receiving co-treatment with either MF or RSV showed significant improvement in the level of these metabolic enzymes when compared with the arsenic exposed group. However when comparing the MF treated group with the RSV treated group, no significant differences in the levels of these metabolic enzymes were observed. These enzymes facilitate the breakdown of polysaccharides (starch) into α-limit dextrin, maltose, and maltotriose, aiding the absorption of carbohydrates from the intestines into the bloodstream [25,32]. Kashtoh and Baek (2022) also reported that one of the most effective ways to lower postprandial hyperglycemia (PPHG) in diabetes mellitus is by inhibiting digestive enzymes, like α-glucosidase and α-amylase, which hydrolyze carbohydrates. This reduces the amount of absorbed glucose. The critical enzyme α-glucosidase is responsible for catalyzing the final phase of carbohydrate digestion. Therefore, α-glucosidase inhibitors can delay glucose absorption, decrease the release of D-glucose from complex carbohydrates, and thereby lower postprandial plasma glucose levels, effectively managing PPHG [33]. MF acts like α-glucosidase inhibitors. Both fasting and postprandial hyper-glycaemia were reduced when metformin was administered. It increases glucose uptake, thereby decreasing appetite. The detailed mechanism of action of MF is given below.

Metformin exerts its mechanism of action by the activation of AMPK, which halts gluconeogenesis process by enhancing hepatic insulin sensitivity. The process of lipogenesis is also inhibited by AMPK induced phosphorylation. This lowers malonyl-CoA production, which activates carnitine palmitoyl-transferase 1 (CPT1) and promotes fatty acid oxidation by improving the import of acyl-CoA into mitochondria. AMPK activation reduces hepatic steatosis and enhances insulin sensitivity, which in turn inhibits gluconeogenesis. Metformin also lowers the post-prandial plasma glucose levels, and may help the body to store glucose as hepatic glycogen after meals. The rapid pace of gluconeogenesis is slowed down by replenishing the glycogen storage, which also lowers the rate of glycogen cycling and hepatic glucose production. Figure 19F gives the detail mechanism of MF. RSV exerts its effect due to the hydroxyl group (as shown in the Figure 19G) interacting with α-glucosidase through hydrogen bonding.

However, we noted a slight increase in serum α-amylase levels (mean = 84.64 ng/mL + 2.330) in sodium arsenate exposed mice compared to the normal or treated group. Glucose-6-phosphatase (Glc-6-Pase) is involved in the hydrolysis of glucose 6-phosphate to glucose, representing the terminal step in both hepatic gluconeogenesis and glycogen breakdown. This plays a pivotal role in hepatic glucose production. Insulin downregulates the gene expression of glucose-6-phosphatase. ADP-arsenate is formed when arsenic interacts with phosphate binding sites in ATP, consequently inhibiting ATP-requiring metabolic pathways [34,35,36]. Arsenic exposure also led to an increase in the gene expression of Glut2 due to changes in DNA methylation of the Glut-2 gene promoter [37]. Furthermore, we observed an upregulation in the gene expression of Glucose transporter-2 (GLUT-2) and glucose-6-phosphatase (G6PC), along with a downregulation in the expression of Glucokinase (GK), all involved in carbohydrate metabolism. The gene expression of GLUT-2 and G6PC was significantly increased in relation to the control group.

**Figure 20:**
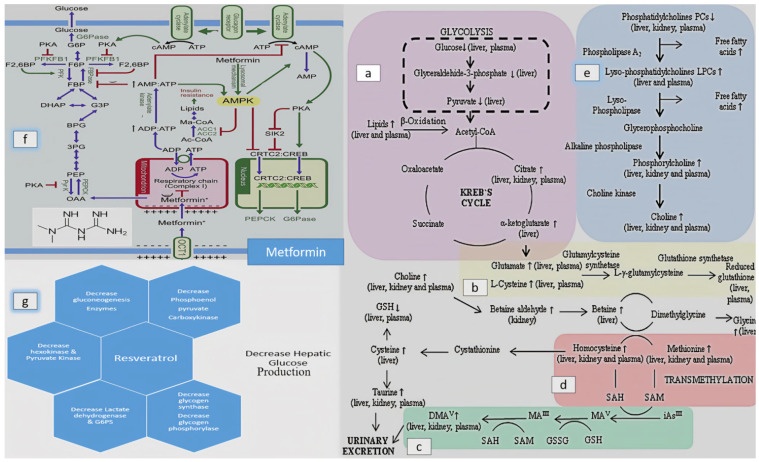
Arsenic induced disruption of metabolic pathway, along with mechanism of MF and possible ameliorating actions of resveratrol. (**a**) Metabolic pathway, (**b**) synthesis of glutathione, (**c**) biomethylation of arsenic, (**d**) alteration in methionine cycle, (**e**) breakdown of phospholipids, (**f**) mechanism of action of metformin and (**g**) resveratrol proposed actions for decrease in hepatic glucose production.

Arsenic binds to thiol-containing active sites of enzymes or interacts with molecules containing sulfhydryl groups, such as cysteine and reduced glutathione (GSH) as shown in Figure 20D. The cellular level of GSH decreases when arsenates are reduced to arsenites, which have a strong affinity for GSH. In this reduction process, GSH acts as an electron donor. Additionally, the level of GSH decreases when free radicals generated by arsenic oxidize GSH. Cell death occurs due to the reduction of cellular ATP caused by the obstruction of the Krebs cycle and disruption of oxidative phosphorylation by arsenic. The interaction of arsenic with thiol groups also inhibits GSH reductase and thioredoxin reductase, contributing to cytotoxicity [38,39]. GSH also serves as a substrate for GPx, which reduces lipid hydroperoxides into lipid alcohols. A commonly studied indicator of oxidative stress is MDA, which provides an indication of the overall level of lipid peroxidation in plasma. Excessive reactive oxygen species (ROS) and reactive nitrogen species (RNS) are major contributors to oxidative stress, leading to cell death and influencing signaling pathways [40,41]. During arsenic metabolism, the activity of SOD decreases due to the production of superoxide. SOD dismutates superoxide anions, preventing the subsequent formation of hydroxyl radicals. The superoxide radical also suppresses the activity of catalase, responsible for converting hydrogen peroxide into water and oxygen [42,43]. The effects of arsenic on the body’s antioxidant defense system seem to be manifested through lipid peroxidation, glutathione depletion, and reduced activity of several enzymes involved in free radical metabolism, including SOD, CAT, GPx, GR, and GST [44]. In our study, after intoxication with sodium arsenate, there was a significant decrease in GSH, GR, GPx, Catalase, and SOD levels reported in sodium arsenate-exposed mice at *p* < 0.05. However, the level of MDA had significantly increased at *p* < 0.05. The levels of GPx, Catalase, and SOD were significantly improved in mice treated either with the standard drug or in the group receiving RSV along with the exposure of arsenic. The ameliorating effect of RSV was comparable with the standard drug and no significant deviation was reported from the control group Both groups showed improved GPx, Catalase, and SOD levels compared to the control group.

RSV enhances detoxification activity of the free radical. It exerts its action either by reduction of ROS production when it competes with coenzyme Q or by activation of peroxisome proliferator, activated receptor gamma coactivator 1-alpha, which induces the expression of various ROS detoxifying enzymes, such as GPX, CAT, and SOD

Elevated levels of C-reactive protein (CRP) have been associated with excess body weight due to the generation of key stimulators of CRP, such as tumor necrosis factor (TNF-) and interleukin 6 (IL-6), by adipocytes [45]. In diabetes mellitus, increased production of CRP by hepatocytes has been reported. The levels of inflammatory biomarkers, namely C-reactive protein and IL-6, were significantly increased (*p* < 0.05) in the group treated with sodium arsenate (Figure 6A,B). In contrast, the group receiving MF or RSV in addition to sodium arsenate showed a significant decrease in the levels of inflammatory biomarkers compared to those in the arsenic-exposed group. Similarly, the levels of kidney biomarkers, such as serum creatinine and blood urea, and liver biomarkers were significantly increased (*p* < 0.05) in the arsenic-exposed group compared to the standard treated and RSV-received group. Rouse et al. in 2013 also reported significant elevations in serum levels of alkaline phosphatase, alanine aminotransferase, and BUN in diabetic rats compared to non-diabetic rats [46].

Arsenic has a broad impact on various disease processes, including cell signaling, cell cycle control, oxidative stress, and DNA repair. Several studies have revealed significant dose, time, and tissue-specific variations resulting from arsenic exposure, as well as significant gene-environment and co-exposure interactions [47,48,49]. Previous research has demonstrated that arsenic affects lipid and amino acid metabolism, which is intricately linked to fatty acid beta-oxidation and amino acid metabolic disorders. Cellular energy production involves the consumption of fatty acids and glucose through the processes of beta oxidation and glycolysis. During beta oxidation, fatty acyl groups are transported from the cytosol through the outer and inner mitochondrial membranes. There are two types of Carnitine palmitoyl transferase (CPT) in mitochondria: CPT I is located in the outer membrane, while CPT II is found in the inner mitochondrial membrane. These enzymes play a crucial role in the esterification of fatty acids in the mitochondrial membrane. Carnitine palmitoyl transferase 1 (CPT1) catalyzes the transport of acyl-CoA from the outer mitochondrial membrane to the mitochondrial matrix in the form of acyl-carnitine. Carnitine palmitoyl transferase II converts acyl-carnitine back to acyl-CoA [50]. Lecithin cholesterol acyltransferase (LCAT) is responsible for esterifying cholesterol to form cholesterol ester (CE) and lysophosphatidyl-choline (LPC) [51]. Carnitine-acylcarnitine translocase (CACT) facilitates the transfer of long-chain fatty acyl-carnitines across the inner mitochondrial membrane [52]. In our study, we observed a significant upregulation in the gene expression of Carnitine Palmitoyl-transferase-I (CPT-I), Carnitine Palmitoyl-transferase-II (CPT-II), Lecithin–Cholesterol Acyl-transferase (LCAT), Carnitine O-Octanoyl-transferase (CROT), and Mitochondrial carnitine/acylcarnitine carrier protein (CACT) responsible for lipid metabolism in the livers of sodium arsenate-exposed mice. This upregulation was significant when compared with the control group or standard treated group, or with the RSV treated group at *p* < 0.05. Additionally, the co-treatment group receiving RSV in addition to sodium arsenate exhibited a significant (*p* < 0.05) decrease in the levels of these genes. However, either the standard drug, i.e., MF, or an RSV could not restore the full gene expression in the presence of continuous exposure of risk factors for the disease.

We have observed a significant upregulation in the gene expression of 5-methyltetrahydrofolate-homocysteine methyltransferase (MTR), which is responsible for amino acid metabolism. The synthesis of the enzyme methionine synthase is guided by instructions encoded in the MTR gene. Methionine synthase plays a crucial role in processing amino acids, which are the fundamental building blocks of proteins. This enzyme facilitates the conversion of homocysteine, another amino acid, into methionine through a chemical reaction it mediates. Arsenic exposure increases the utilization of methionine and S-adenosylmethionine (SAM) for arsenic methylation, as shown in Figure 19D. Methionine adenosyl-transferase is responsible for converting methionine into SAM, which then interacts with arsenic to form methylated and dimethylated arsenic compounds by donating a methyl group [53].

We also reported the altered level of various amino acids, such as Taurine, Threonine, Methionine, Isoleucine, Leucine, Arginine, Lysine, L-Methionine sulfoxide, and Glycine, in arsenic exposed mice when analyzed with an amino acid analyzer (Figure 9). The ms/ms spectra in Figure 16 and Figure 17 also reported the presence of these amino acids in arsenic exposed mice). Decrease in taurine levels cause disturbance in many important metabolic roles, such as modulation of calcium dependent cell signaling, osmoregulation and membrane stabilization. It also plays a role in protection of the cell because of its antioxidant activity. A decreased level of taurine also leads to obstruction of production of GSH from cystein. The arsenic exposure also disturbs the pathway of methionine and homocysteine. (Figure 20). The reduction in arginine, L-methionine and glycine levels is also due to increased biosynthesis of creatinine. (Figure 5 explains the significant high production of creatinine).

Santiago et al. (2019) reported three classes of lipids that were significantly altered by arsenic exposure: glycerophospholipids (lysophospho-lipids and phosphatidylcholines), glycerolipids (triglycerides), and sterol lipids (3-deoxyvitamin D3) [19]. Lysophosphatidyl-cholines are produced through free radical-catalyzed oxidation of polyunsaturated phosphatidylcholines. Overstimulation of phospholipase A results in the breakdown of the PC membrane and subsequent accumulation of LysoPCs under pathological situations. LysoPCs can also be generated in plasma through the action of lecithin-cholesterol acyltransferase (LCAT). Wang and colleagues observed a significant increase in LysoPCs and increased expression of hepatic LCAT in male rats treated with 10 ppm arsenic, suggesting that arsenic exposure may disrupt the transformation process of LysoPCs, leading to their accumulation [54]. Various metabolites were identified as lipid metabolites (such as L-carnitine, sphinganine, phyto-sphingosine, and lysophosphatidyl-choline), amino acids, and arsenic metabolites [18,55]. Lipid metabolites (57 metabolites in MS/MS ESI+ mode and 75 metabolites in MS/MS ESI− mode) were primarily associated with the disruption of important metabolic pathways. These lipids metabolomes belongs to various classes of lipids, like fatty acids/esters (FA), tri(acyl|alkyl)-glycerols (TG), glycero-phosphocholines (PC), glycerophosphates (PA), glycero-phosphoserines (PS), glycero-phosphoethanolamines (PE), glycero-phosphoglycerols (PG), glycero-phosphoinositols (PI), sphingolipids, like Hexosyl ceramides (HexCer) and Ceramide phosphates (CerP), and sterol lipids such as Sterols and cholesterol esters (ST and CE). Arsenic induced apoptosis cause increased levels of FFAs, TGs, diacylglycerols in plasma. Many pathological mechanisms and crucial cellular functions, such as signal transduction, apoptosis, protein sorting and necrosis are associated with phosphotidyl-cholines, which undergo enzymatic degradation with phospholipase- 2 and release one of the fatty acid groups. The choline and phosphorylcholine were produced by the hydrolysis of lyso-phosphatidylcholine (LPC). LPS causes cell apoptosis, in which breakdown of membrane phospholipids occurred (explained in Figure 20D), which was also evident from the presence of arachidonic acid, linoleic acid and gamma linoleic acid in the serum of arsenic exposed mice (Figure 18) This breakdown also caused the accumulation of choline in plasma and tissue. This leads to perturbations of the choline degradation pathway. Betaine is produced by the oxidation of choline, which causes the methylation of inorganic arsenic (Figure 20C).

Metabolomic analysis identified lipid metabolomes, amino acids, and arsenic metabolites, revealing their involvement in the disruption of essential metabolic pathways. For instance, Diacylglycerol induces insulin resistance through activation of Protein kinase C epsilon type enzyme (PKC-ε) and decreased insulin receptor (IRS-2) tyrosine phosphorylation. Ceramide is a key molecule in sphingolipid metabolism, insulin resistance, and metabolic dysregulation [56,57,58,59]. Glycerophospho-cholines contribute to insulin resistance (IR) and glucose intolerance [60,61]. These effects were supported by histopathological changes observed, including distention and hemorrhage in the central and portal veins, loss of normal architecture, degenerated hepatocytes with pyknotic nuclei, vacuolated hepatocytes, dilation of blood sinusoids, and an increased number of Kupffer cells in the livers of arsenic-exposed mice [62,63].

## 5. Conclusions

This study aimed to comprehensively investigate the biochemical profiles and metabolomics associated with arsenic-induced metabolic disorders, focusing on disruptions in lipid, carbohydrate, and amino acid metabolism. Additionally, we evaluated the therapeutic potential of resveratrol (RSV) in mitigating arsenic-induced diabetes. Our findings underscore the ameliorative effects of resveratrol in alleviating the consequences of arsenic intoxication. Our results demonstrated notable improvements due to resveratrol treatment in the context of arsenic exposure. We assessed various biochemical biomarkers, including glycemic control indicators (serum glucose, insulin levels, HbA1c, and HOMA-IR), carbohydrate metabolism markers (amylase, hexokinase, glucosidase, and G6PC), liver function (ALT and AST), kidney function (blood urea nitrogen (BUN) and creatinine), oxidative stress markers (malondialdehyde (MDA), superoxide dismutase (SOD), and glutathione), catalase levels, and inflammatory biomarkers (IL-6, CRP). Quantitative real-time PCR (qRT-PCR) analysis of gene expression further corroborated the impairment of lipid metabolism (LCAT, CROT, CPT-1, CPT-II, CACT), amino acid metabolism (MRT), and carbohydrate metabolism (GAPDH, GK, GLUT-2, G-6-PASE). Moreover, serum metabolomics analysis using MS/MS provided additional insights into the mechanisms through which lipid metabolism, amino acid metabolism, and carbohydrate metabolism were adversely affected in the arsenic-exposed group, reminiscent of diabetes mellitus. These comprehensive findings shed light on the intricate interplay between arsenic exposure and disrupted metabolic pathways, emphasizing the potential of resveratrol as a therapeutic strategy to mitigate these harmful effects.

In conclusion, this study delved into the intricate biochemical and metabolomics alterations induced by arsenic exposure, particularly highlighting disruptions in lipid, carbohydrate, and amino acid metabolism. The investigation revealed significant perturbations in various biomarkers associated with diabetes mellitus and metabolic dysfunction. Notably, our findings unveiled the promising therapeutic potential of resveratrol in mitigating the adverse effects of arsenic intoxication.

The outcomes of this study not only underscore the deleterious effects of arsenic exposure on metabolic pathways but also emphasize the importance of interventions aimed at restoring metabolic balance. The potential of resveratrol as a therapeutic agent for counteracting arsenic-induced metabolic disorders holds promise for clinical applications. Moving forward, these findings contribute to our understanding of the intricate relationship between environmental toxins, metabolic disruptions, and potential remedies, paving the way for further research and interventions to safeguard metabolic health in the face of toxic exposures.

## Figures and Tables

**Table 1:** Primer sequences employed for the qRT-PCR analysis of the targeted genes related to lipid metabolism, amino acid metabolism, and carbohydrate metabolism.

Metabolism	Gene Name	Primer	Primer Sequence	Size (bp)
Lipid metabolism	Beta-actin	Forward	5’-CCCATCTATGAGGGTTACGC-3‘	150
		Reverse	5’-TTTAATGTCACGCACGATTTC-3‘	
	Carnitinepalmitoyl- transferase I (CPT-I)	Forward	5′-ATCCACCATTCCACTCTGCT-3′	107
		Reverse	5′-TGTGCCTGCTGTCCTTGATA-3′	
	Carnitinepalmitoyl transferase II (CPT-II)	Forward	5′-CTGTCCACCAGCACTCTGAA-3′	111
		Reverse	5′-GCAACCTATCCAGTCATCGT-3′	
	Lecithin–cholesterol acyltransferase (LCAT)	Forward	5′-CTCCTTCTGGCTCCTCAATG-3′	171
		Reverse	5′-TCCTCTGTCTTTCGGTAGCAC-3′	
	Carnitine O-octanoyl6transferase (CROT)	Forward	5′-AGACGGAAGGGAGATGGAG-3′	168
		Reverse	5′-AAGATGTGAAGGTAGATGCTGCT-3′	
	Mitochondrial carnitine/acylcarnitine carrier protein (CACT)	Forward	5′-TTCTCCACTGCTGCTCCTG-3′	100
		Reverse	5′-CCTGTCTGCTCCCATTCAG-3′	
Amino acid metabolism	5-methyl tetrahydrofolate-homocysteine methyltransferase (MTR)	Forward	5′-GGTTCGGTTGAAGAAGAGGA-3′	112
		Reverse	5′-TATTACAGCCCAGCACCACA-3′	
Carbohydrate metabolism	Glyceraldehyde-3-phosphate dehydrogenase (GAPDH)	Forward	5′-CCCGTAGACAAAATGGTGAAGGTC-3′	215
		Reverse	5′-GCCAAAGTTGTCATGGATGACC-3′	
	Glucose transporter-2 (GLUT-2)	Forward	5′-TTAGCAACTGGGTCTGCAAT-3′	243
		Reverse	5′-TCTCTGAAGACGCCAGGAAT-3′	
	Glucokinase (GK)	Forward	5′-CACCCAACTGCGAAATCACC-3′	162
		Reverse	5′-CATTTGTGGGGTGTGGAGTC-3′	
	Glucose 6 Phosphate (G6PH)	Forward	5′-AAAGAGACTGTCGGCATCAATC-3′	
		Reverse	5′-AAGAGGCTGGCAAAGGGTGTAG-3′	

**Table 2:** Instrument parameters for MS/MS.

Parameter	Particulars
Instrument with model	Linear Ion Trap Mass spectrometer LTQ XL (Thermo Electron Scientific, Waltham, MA, USA) equipped with electro spray ionization source
Software	Xcalibur 2.0.7
Fragmentation (MS/MS)	Various peaks were selected for fragmentation, using collision-induced dissociation (CID) energy ranging from 20–30
Mode of ionization	Both negative and positive scan ion modes
Mode of injection	Direct insertion method
Scanning mass range	50–2000 *m*/*z*
Sheath gas flow rate	17 units
Auxiliary gas flow rate	6 units
Solvent	Methanol
Flow rate	9.8 μL/min
Capillary voltage	4.7 kV
Capillary temperature	278 °C

**Table 3:** Metabolites identified in MS/MS spectra of arsenic exposed mice serum.

Metabolites	Formula	M.wt.	Ion	Precursor Ion (*m*/*z*)	Product Ion (*m*/*z*)
Lipid metabolomes					
Sphinganine	C_18_H_39_NO_2_	302	[M+H]+	303	285, 190.3, 176.3, 150, 119.92 and 106.
Phytosphingosine	C_18_H_39_NO_3_	317	[M+H]+	318	300 and 256
Lyso phosphatidylcholine	C_26_H_54_NO_7_P	523	[M+H]+	546	487, 341, 404, and 443
Carnitine	C_7_H_16_NO_3_	162	[M-H]^−^	160.75	162.92,102.92,84.92 and 60.08
Amino acid					
Arginine	C_6_H_14_N_4_O_2_	174.20	[M+H]+	175.08	130.08,116.00,70.08 and 60.08
Dimethyl Arginine	C_8_H_18_N_4_O_2_	202.25	[M+H]+	203.00	185.00,158.17,116 and 70.00
Isoleucine	C_6_H_13_NO_2_	131.17	[M+H]+	132.00	86.08 and 58.08
Valine	C_5_H_11_NO_2_	117.14	[M+H]+	118	72.08
Thronine	C_4_H_9_NO_3_	119.11	[M+H]+	120	74.08

**Table 4:** Identified Arsenic Metabolites in the MS/MS Spectra of Serum from Arsenic-Exposed Mice.

Inorganic Arsenic with Its Metabolites	Ion	Molecular Ion Structure	Molecular Ion *m*/*z*	Characteristic Fragments	Fragment Ion Structure
Arsenate (As^V^)	[M-H]^−^	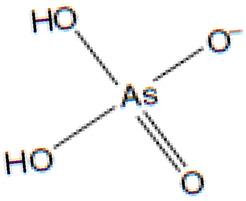	141	123	AsO_3_^−^
Arsenite (As^III^)	[M-H]^−^	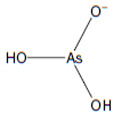	125	109	As(OH)_2_^−^
Monomethyl arsenic acid (MMA^V^)	[M-H]^−^	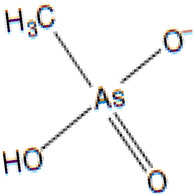	139	125 123 121 107	AsO_3_H− AsO_2_CH_2_^−^ AsO_2_^−^
Dimethylarsi-nous acid (DMA^III^)	[M-H]^−^	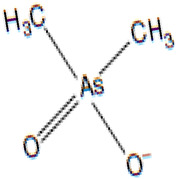	137	107, 122	AsO_2_^−^ CH_3_AsO_2_^−^

## Data Availability

All data are available in this manuscript.
